# The kinase activity of integrin-linked kinase regulates cellular senescence in gastric cancer

**DOI:** 10.1038/s41419-022-05020-3

**Published:** 2022-07-01

**Authors:** Chengbo Ji, Mili Zhang, Junjie Hu, Can Cao, Qisheng Gu, Youdong Liu, Xu Li, Duogang Xu, Le Ying, Yuqin Yang, Hugh Gao, Jikun Li, Liang Yu

**Affiliations:** 1 Department of General Surgery, Shanghai General Hospital, Shanghai Jiao Tong University School of Medicine, Shanghai, 201620 China; 2 Department of General Surgery, Zhongshan Hospital, General Surgery Research Institute, Fudan University, Shanghai, 200032 China; 3 Department of Gastroenterology, Shanghai General Hospital, Shanghai Jiao Tong University School of Medicine, Shanghai, 201620 China; 4 Institut Pasteur of Shanghai, Chinese Academy of Sciences, Shanghai, 200031 China; 5 Centre for Innate Immunity and Infectious Diseases, Hudson Institute of Medical Research, Clayton, VIC 3168 Australia; 6 Department of Molecular Translational Science, Faculty of Medicine, Nursing and Health Sciences, Monash University, Clayton, Victoria 3800 Australia; 7 Department of Laboratory Animal Centre, Shanghai General Hospital, Shanghai Jiao Tong University School of Medicine, Shanghai, 201620 China

**Keywords:** Gastric cancer, Senescence

## Abstract

The activity of integrin-linked kinase (ILK) in cancerous cells is often oncogenic and associated with malignant properties, such as uncontrolled cell cycle progression and evasion from senescence. However, the role of ILK in cellular senescence in gastric cancer (GC) has not been previously examined. We generated single-cell clones of ILK knock-out using CRISPR-Cas9 in human GC lines with mesenchymal or epithelial histology. Cells with no residual ILK expression exhibited strong cellular senescence with diminished clathrin-mediated endocytosis, Surprisingly, ILK loss-induced cellular senescence appeared to be independent of its function in integrin signaling. The low dose of CPD22, a small molecule inhibitor of ILK activity-induced senescence in three GC cell lines with different histologies. Furthermore, senescent cells with ILK depletion transfected with N-terminal truncated ILK mutant remaining catalytic domains displayed the reduction of senescent phenotypes. RNA sequencing and cytokine array results revealed the enrichment of multiple pro-inflammatory signaling pathways in GC lines in the absence of ILK. Our study identified the important role and the potential mechanism of ILK in the cellular senescence of cancerous epithelial cells. The inhibition of ILK activity using small molecule compounds could have a pro-senescent effect as a therapeutic option for GC.

## Introduction

Integrin-linked kinase (ILK) was discovered as a unique intracellular adaptor that links integrins and various adhesion molecules to the regulation of the actin cytoskeleton and cellular signaling cascades [1, 2]. By analyzing its binding partners, such as PINCH1, PARVINS, and paxillin, ILK has been identified as an essential scaffolding point for focal adhesion assembly [3, 4]. Loss of ILK resulted in the incorrect subcellular localization of many molecules and disruption of cytoskeleton organization [2]. ILK was also described as an untypical serine/threonine-protein kinase, activated by integrin-mediated cell adhesion and PI3K activity. Previous studies indicated that two downstream targets of PI3K, phosphorylation of AKT at Ser 473 and GSK3β at Ser 9, were regulated by cytoplasmic ILK signaling transduction [5], which have been implicated in cell survival, anoikis, and proliferation. ILK expression or activity was often elevated in various human cancers, such as stomach, pancreas, colon, and malignant melanoma [2]. As its direct and functional linkage to integrins and cytoskeleton, oncogenic ILK signaling links to initiating epithelial-mesenchymal transition and promoting highly migratory or invasive properties [6, 7].

However, the roles of ILK in cellular senescence remain contextually elusive [8]. For example, earlier studies showed that in primary cardiac fibroblasts from young rats, overexpression of ILK induced various cellular senescent phenotypes, such as enlarged cell shapes, lower proliferation rate, and increased enzymatic β-galactosidase activity [9]. Genetic reduction of ILK in *C. elegans* led to lifespan extension without majorly affecting cytoskeletal integrity [10]. In contrast, cyclin D1 expression and CDK4 activity were elevated in the suspension cultures of ILK-overexpressing rat intestinal epithelial cell (IEC), along with hyper-phosphorylation of the retinoblastoma protein and an increased proportion of cells in S phase [11]. It has also been documented that ILK regulates cyclin D1 expression, cell proliferation, and morphology in other normal cell types, such as chondrocytes and endothelial cells [12, 13]. There are fewer studies of ILK in cellular senescence for cancerous cells. Persad et al. demonstrated that the activity of ILK is constitutively elevated in a serum- and anchorage-independent manner in PTEN-mutant cells [14]. The inhibition of ILK by transfection of dominant-negative ILK results in suppression of cyclin D1 expression and G1/S cell-cycle arrest in prostate cancer cells [14]. Alternatively, anti-ILK therapies may have different outcomes in cancerous cell lines that depend on retinoblastoma gene expression [15].

Here, we investigated the role of ILK in senescence in gastric cancer (GC) by generating ILK-depleted single-cell clonal populations from two human gastric epithelial cancer lines (MKN1 and MKN28) with different histological phenotypes. Based on the data in vitro and in vivo, we showed that the loss of ILK in GC cells induces cellular senescence, characterized by increased activity of lysosomal enzyme, G1/G2 cell cycle arrest, and metabolic alteration in a p53/p21 dependent manner. These observations were at least partially traced to increased AMPH (Amphiphysin) cleavage and dynamin II instability, resulting in the reduction of clathrin-mediated endocytosis (CME). Significantly, ILK regulated cellular senescence in GC lines in a kinase activity-dependent manner. The low dose of CPD22, a small molecule inhibitor of ILK activity, was sufficient to induce both line's senescence without influencing focal adhesion dynamics and cytoskeleton remodeling. In tandem with these data, only an N-terminal truncated ILK variant remaining catalytic domains rescued senescent phenotypes in MKN1 cells lacking ILK. Coupling with the results from RNA sequencing (RNA-seq) and cytokine arrays, we also revealed that ILK knock-out led to increased multiple pro-inflammatory signaling pathways in GC lines. Therefore, the blockade of ILK activity could be a potential therapeutic option for GC.

## Materials and methods

### Cell culture

Human GC cell lines MKN1, MKN28 (Japanese Collection of Research Bioresources Cell Bank), and AGS (American Type Culture Collection, ATCC) were cultured in RPMI 1640 medium supplemented with 10% fetal bovine serum (FBS) and 1% L-glutamine (Gibco). Lenti-X 293 T cells (Clontech) were cultured in DMEM supplemented with 10% FBS. Cell line identification was authenticated by short-tandem repeat profiling (PowerPlex HS16 System Kit; Promega). Cells were routinely tested for mycoplasma contamination (MycoAlert PLUS Mycoplasma Detection Kit; Lonza). Cells were incubated in a 5% CO_2_ incubator at 37 °C.

### CRISPR-driven gene editing

Self-complementary oligonucleotides (NovaBio) used as single-guided (sg) RNA sequences for targeting human ILK (exon 2, sgRNA11, ACGCAGTCGCCGTTCGCCTG) and non-target control (sgRNA1, ACGGAGGCTAAGCGTCGCAA) were ligated into the LentiCRISPRv2 construct (Addgene). Lentivirus was produced by transfecting vectors into Lenti-X/H293T cells with LentiCRISPR: psPAX2: pMD2.G at a ratio of 4:3:1. The virus was harvested 48 h after transfection, filtered, and added into cultures containing 5 mg/mL polybrene, followed by spinoculation. Infected cells were selected with puromycin, and the generation of single-cell clonal population with ILK depletion was conducted in 96-well plates by serial dilution. Clonal populations with no residual ILK were selected and amplified for various experiments.

### SA-β-Gal assay

Cells were washed with phosphate-buffered saline (PBS), fixed with 3% formaldehyde for 5 min, and washed with PBS. Cells were then incubated in SA-β-Gal staining solution (Beyotime Biotechnology) overnight at 37 °C. Cellular senescence was scored as a percentage of SA-β-Gal–positive cells (blue staining) relative to the total cell number. Tissues were flash-frozen in liquid nitrogen and embedded in optimal cutting temperature compounds. Tissues were immediately cut into 4-μm sections, fixed with 1% formaldehyde in PBS, and incubated in SA-β-Gal staining solution overnight at 37 °C. After incubation, the nuclei were stained with Safranin-O.

### Immunoblotting

The cell lysates were extracted using RIPA lysis buffer with Protease and Phosphatase Inhibitor Cocktail (NCM Biotech). Cell lysates were electrophoresed on 10–12% SDS-PAGE gels followed by transferring to PVDF membranes (Millipore). Total protein lysates were immunoblotted with antibodies against ILK, phospho-AKT (Ser473), total AKT, phospho-GSK3β, total GSK3β, p21 Waf1/Cip1, p16 INK4A, phosphor-Rb (Ser807/811), total Rb, phospho-p53 (Ser15), total p53, phospho-FAK (Tyr397), total FAK, phospho-Paxillin (Tyr118), total Paxillin (Cell Signaling Technologies), GAPDH, α-PARVIN, β-PIX (ARHGEF7), Amphiphysin (Proteintech), Dynamin II (Santa Cruz). Protein bands were visualized and analyzed using the Odyssey CLX Infrared Imaging System (LI-COR) and Tanon-5200 Chemiluminescence Imaging System. The protein expression was quantified by densitometry and normalization to GAPDH expression levels with ImageJ software (Version 1.8.0). Original data was shown in a single document in supplementary files.

### In vivo mouse models

For tumor xenograft model, 4-week-old female NOD.Cg-Prkdc^scid^ Il2rg^tm1Wjl^/SzJ/Arc (NSG) mice were used. All NSG mice were injected into the right flank subcutaneously with 2.0 × 10^6^ MKN1 NTC cells or ILK-KO cells to establish a xenograft model. Each group had five mice and tumor volumes were measured every 3 days. All mice were sacrificed after 5 weeks or the end point of the experiment, and tumor weights were measured after sacrificing mice.

### Immunohistochemistry staining

Tissues were fixed with 10% neutral buffered formalin, dehydrated, and embedded in paraffin. Sections (4-μm thick) were cut from formalin-fixed, paraffin-embedded tissue blocks. After deparaffinization, slides were subjected to an antigen retrieval procedure in 10 mM sodium citrate buffer (pH 6.0) for 3 min using a microwave. Slides were incubated in methanol containing 0.3% hydrogen peroxide for 20 min at room temperature to block endogenous peroxidase activity before applying the blocking solution. In all, 5% BSA solution was used as a blocking buffer. Then slides were incubated with primary antibodies at 4 °C overnight. Immunohistochemistry staining (IHC) was performed with primary antibodies against p-γH2AX (1:450 dilution), ILK (1:100 dilution) (Cell Signaling Technology), p21 Waf1/Cip1 (1:200 dilution), p16 INK4A (1:200 dilution), Amphiphysin (1:200 dilution) (Proteintech), PCNA (1:2000 dilution), and p53 (1:500 dilution) (Servicebio), along with concentration matched isotype control (Cell Signaling Technology). Slides were then incubated with a biotin-conjugated secondary antibody for 30 min, and followed peroxidase-conjugated streptavidin incubation for 30 min at room temperature. Peroxidase activity was detected using the substrate DAB. For histologic evaluation, sections were stained with hematoxylin and eosin. The percentage of different antibodies positive cells were displayed by IHC scores (intensity×number, Intensity: 0, none; 1, mild; 2, moderate; 3, strong; 4, severe. Number: 1, 0%~25%; 2, 25%~50%; 3, 50%~75%; 4, 75%~100%).

### Immunofluorescence staining

Cells were plated in confocal dishes for two days followed by fixation in 4% paraformaldehyde at room temperature for 20 min. After permeabilization with 0.1% Triton X-100 for 5 min, cells were incubated in 1%BSA in PBS for 1 h at room temperature. Cells were then incubated overnight with primary antibodies against ZO-1 (1:500, Proteintech) followed by incubation with goat anti-Rabbit Secondary Antibody, Alexa Fluor 488 (1: 5000, Invitrogen), and TRITC-conjugated Phalloidin (1:1000, Sigma-Aldrich). Staining of focal adhesion was conducted with the Actin Cytoskeleton and Focal Adhesion Staining Kit (Merck Millipore). For antigen staining, cells were incubated with anti-vinculin primary antibody (1:200, Merck Millipore) overnight at 4 °C, followed by incubation with a secondary FITC-conjugated antibody (1:5000, Proteintech) and TRITC-conjugated Phalloidin (1:1000, Sigma-Aldrich) for 1 h, respectively. Nuclei counterstaining was performed by incubating cells with DAPI for 5 min at room temperature. The quantification of focal adhesion (FA) number, actin and ZO-1 intensity were determined using ImageJ software (Version 1.8.0). Original data were displayed in supplementary data.

### Cell proliferation and soft agar colony-forming assays

MKN1, MKN28, and AGS cells were seeded in triplicates in 96-well plates. Cell proliferation was assessed with the Cell-Light EdU Apollo567 In Vitro Kit (RiboBio). For soft agar colony formation assay, cells were suspended in RPMI 1640 containing 0.3% BD Difco Agar and 10% FBS, and suspensions layered on RPMI 1640 containing the same 0.4% agar and 10% FBS in triplicates in 6-well plates. Colonies were photographed at day 20–22 after plating. EdU positive cells and soft agar colonies were counted by ImageJ software (Version 1.8.0).

### Quantitative real-time PCR

Total RNA was isolated from human GC cell lines using RNeasy Mini Kit (Qiagen) with DNase treatment (Qiagen). RNA was then transcribed using the PrimeScript RT Master Mix (TAKARA). Quantitative real-time PCR (qPCR) was performed using QuantStudio™ 6 Flex System (ThermoFisher). Gene expression was normalized to the expression of housekeeping gene 18 S. Relative fold changes were transformed using the comparative threshold cycle (CT) method (the 2^−ΔΔCT^ method) with the QuantStudio™ Real-Time PCR Software. Primer sequences are provided in Supplementary Table S1.

### Bioenergetic assays

Real-time oxygen consumption rate (OCAR) and extracellular acidification rate (ECAR) were measured using an XFe-96 Extracellular Flux Analyzers (Seahorse Bioscience, Agilent). MKN1 and MKN28 were plated at 1.5 × 10^4^ per well. AGS cells were plated at 1.0 × 10^4^ per well. On the day of assay, the normal RPMI 1640 medium was changed into Seahorse assay medium with 1 mM pyruvate, 2 mM glutamine, and 10 mM glucose (adjust the pH to 7.4) for Mito Stress assay and 2 mM glutamine for Glycolysis Stress assay. Then cells were incubated in a 37 °C non-CO_2_ incubator for 1 h prior to assay. Pharmaceutical compounds in the assay kit were used at the indicated concentration (Mito Stress: oligomycin, 1 μM; FCCP, 0.2 μM for MKN1, 0.5 μM for MKN28 and AGS, antimycin A and rotenone mix 0.5 μM. Glycolysis Stress: glucose, 10 μM; oligomycin, 1 μM; 2-DG 10 μM).

### L-lactate assay and ROS measurement

The production of lactate was analyzed to determine the rate of aerobic glycolysis. l-lactate was measured by l-Lactate Assay Kit (Colorimetric) (Abcam) following the manufacturer’s instructions. The lactate production was assessed by measuring the absorbance at 450 nm and calculated using the equation obtained from the linear regression of the standard curve. Total cellular ROS was measured using the ROS detection assay kit (Abcam) following the kit protocol. The assay was incubated for 60 min at 37 °C in dark. Both of the assays were measured on the VarioskanFlash microplate reader (Thermo).

### Flow Cytometry

For cell cycle analysis, cells were starved overnight and stimulated for 10% FBS for 48 h. Cells were collected with trypsin and fixed in 70% ethanol at 4°C overnight. The cells were incubated with Fxcycle PI/RNase Staining solution (ThermoFisher) for 30 min in dark. Flow cytometry was conducted by BD Accuri^TM^ C6 plus workstation and analyzed by Modfit LTsoftware (Version 5.0). For cell apoptosis, Annexin V-APC/7-AAD apoptosis kit was used, and cell staining was followed by the instruction protocol. Flow cytometry for apoptosis was also conducted by BD Accuri^TM^ C6 plus workstation and analyzed by FlowJo software (Version 10).

### RNA sequencing

Twenty ng of each total RNA sample was sequenced in-house using Illumina NextSeq 550 High output mode and v2.5 chemistry following Illumina protocol 15046563 v04. RNA library preparation and sequencing were performed using 19 bp forward read and 72 bp reverse read with up to 400 M reads per run. FASTQ files were demultiplexed into each sample based on the 8 bp sample index in the forward read. Sample index sequences within 1 hamming distance of the expected sample indexes were included. STAR aligner was used to align the reverse reads to GRCh38. UMI deduplication was performed using the Umi-tools package. Aligned gene counts were analyzed using the edge R package (Bioconductor release version 3.32.1) in R (version 4.0.3). Raw counts were converted to counts per million (CPM), and trimmed mean of M-values (TMM) was performed to normalize the data for the differential expression analysis. The heatmap R package was used for scaling and visualization of the RNA-seq data. Hallmark gene sets were downloaded from The Molecular Signature Database (MSigDB, version 7.2) and used for gene set enrichment analysis (GSEA, version 4.0.0).

### Cytokine array and ELISA

Cell supernatants from different clones of MKN1 and MKN28 cells were tested for protein secretion by Human XL Cytokine Array Kit following the manufacturer’s instructions (R&D Systems). For ELISA, all the cytokines were tested in cell supernatant from cell lines mentioned above by using ELISA kit following the manufacturer’s instructions (Multiscience Biotech).

### EGF endocytosis assay

Cells were seeded in a 96 well-plate the day before. Cells were then incubated on ice for 15 min and were washed with cold live cell imaging solution (LCIS) (140 mM NaCl, 20 mM HEPES, 2.5 mM KCl, 1.8 mM CaCl_2_, and 1.0 mM MgCl_2_ (pH 7.4). Cells were incubated with pHrodo™ Red Epidermal Growth Factor (EGF) Conjugate (ThermoFisher) (5 μg/ml) in LCIS containing 20 mM glucose and 1% BSA for 30 min at 37 °C, and were washed three times with LCIS (at 4 °C). Fluorescent labels that respond to a pH change become brightly fluorescent in the acidic endosomes through EGF endocytosis, which allows the measurement of internalized EGF, as well as the dynamic monitoring of EGF internalization in live cells. Cells were fixed with 4% paraformaldehyde in PBS for 10 min, and stained with Hoechst 33342 solution (ThermoFisher) for fluorescence imaging using a ×10/20 objective lens.

### LDL uptake assay

Cells were plated at a density of 4000 cells/well in 96-well plates and cultured in medium supplemented with 10% FBS for 24 h. Then, the culture medium was replaced with 100 µl LDL-DyLight™ 550 solution (1:100) from LDL Uptake Assay Kit (Cell-Based) (Abcam), and cells were incubated for 4 h at 37 °C. Cells were then fixed with 100 µl/well of Cell-Based Assay Fixative Solution for 10 min, were washed with TBST [0.1%Triton X-100, TBS] three times for five minutes each and stained with Hoechst 33342 solution (ThermoFisher) for fluorescence imaging.

### Reagents, transient transfection and construction of mutagenesis

ILK inhibitor Cpd22 (Compound 22) was purchased from Merck Millipore. The IC_50_ value was determined by the Cell Counting Kit-8 (CCK-8) assay (Dojindo Laboratories) in a 96-well plate according to the manufacturer’s instructions. RGD peptide (GRGDNP) (TFA) was purchased from MCE and used at 100 μM. Fibronectin (sigma) was coated at 5 μg/well. Calpain II inhibitor, ALLM (Abcam) was used at 300 nM. Transfection of DNA was performed using Lipofectamine 3000 reagent (Thermo Fisher) following the manufacturer’s instructions. Cells seeded in 12-well and 96-well plates were then transfected with the indicated DNAs and used at 3–4 days using Lipofectamine 3000 (Thermo fisher). Various ILK mutations were generated as previously described [7] and confirmed by Sanger DNA sequencing before experiments.

### Statistical analysis

All data were shown from three independent experiments and presented as the mean ± SD. Statistical comparisons were determined using unpaired student’s *t* test in GraphPad Prism (Version 7.0), and *p* value <0.05 was considered statistically significant.

## Results

### ILK KO induced cellular senescence in GC cancer cells in vitro and in vivo

**Fig. 1:**
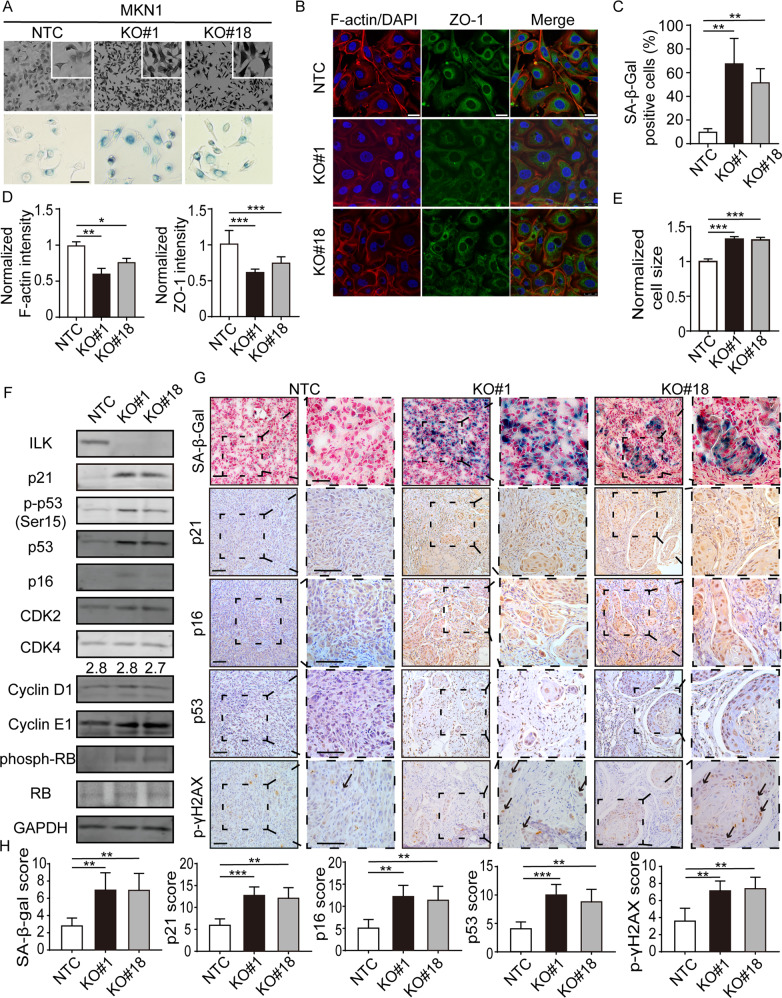
ILK KO induced cellular senescence in MKN1 in vitro and in vivo. **A** Crystal violent staining under microscopy of MKN1 NTC cells, ILK knock-out single-cell clone #1 and #18 with SA-β-Gal staining respectively. Scale bar, 50 μm. **B** F-actin (red)/DAPI (blue) and ZO-1 (green) staining of three selected clones. Scale bar, 25 μm. **C** The percentage of cells with positive staining for SA-β-Gal from **A**. **D** Quantification of F-actin and ZO-1 fluorescent intensity from **B**. **E** Flow cytometry analysis of cell size from indicated clones. **F** Western blot analysis of several cell cycle-related proteins in the lysate of the three clones, with GAPDH as the loading control. **G** Representative pairs of low power (left, solid line) and high power (right, dotted line) photomicrographs images of xenografic tumors that were subjected to SA-β-Gal, p21, p16, p53 and p-γH2AX staining were shown. Scale bar, 200 μm (solid line), 100 μm (dotted line). Arrows, nucleus-staining zone. **H** The percentage of cells stained positively in the field for SA-β-Gal, p21, p16, p53, and p-γH2AX were shown by IHC scores. Data represent the mean ± SD of at least three independent experiments. *p < 0.05, **p < 0.01 and ***p < 0.001.

We first assessed the activity of the lysosomal enzyme SA-β-galactosidase (β-Gal), which has been commonly used as a surrogate marker for cellular senescence. We generated two single- cell clones with ILK knock-out (KO) using targeted sgRNAs for MKN1 and MKN28 with distinct histological phenotypes. As shown in Fig. 1A–C, the SA-β-Gal activity of KO lines (#1 and #18) for MKN1 had significantly increased two to three folds compared to that of non-target control cells (NTC). The crystal violet staining of this mesenchymal-like GC line displayed a more fibroblast-like morphological change in ILK depleted cells (Fig. 1A–C, Supplementary Fig. S1A and S1C). The alteration of cellular polarization was also confirmed by immunofluorescence of ZO-1, a tight junction marker and F-actin in MKN1 cells, with a marked reduction of intensity. The morphological changes were coincided with a significant but mild increase of cell size in ILK KO cells compared to that of NTC (Figs. 1B, D, E, Supplementary Fig. S1B, D, SE). Moreover, loss of ILK in these cells coincided with hyper-phosphorylated RB, increased protein expression of p53 and its Serine 15 phosphorylation, as well as a CDK inhibitor p21CIP (*CDKN1A*, hereafter p21) and p16INK4a (*CDKN2A*), which have been identified as the key controllers of the cell cycle arrest during cellular senescence (Fig. 1F). In contrast, the expression of CDK2, CDK4, and Cyclin proteins was not significantly changed in MKN1 cells (Fig. 1F). These results except the expression of CDK4 were invariably observed in another GC line, MKN28, a cell line with an epithelial-like phenotype (Supplementary Fig. S1F). In addition, immunohistochemistry of formalin-fixed paraffin-embedded (FFPE) sections from cell-derived xenografts showed that tumor-bearing ILK KO cells consistently exhibited increased SA-β-Gal staining, nuclear expression of p21, p16, p53, and pγH2AX, which facilitates specific DNA repair complexes during DNA damage (Fig. 1G, H). Collectively, these data demonstrated that ILK may act as an anti-senescence protein in two GC lines with distinct histology.

### Cells lacking ILK displayed significant G1/G2 cell cycle arrest

**Fig. 2:**
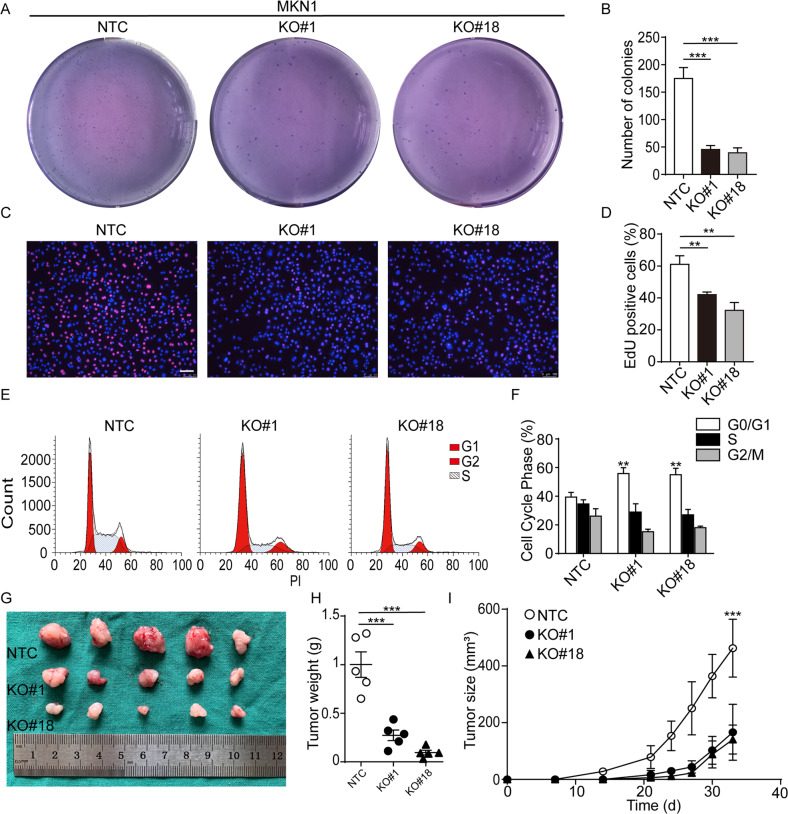
Lack of ILK in MKN1 displayed significant G1/S cell cycle arrest. The proliferation of three MKN1 clones as indicated was measured by soft agar assays **A** and EdU assays **C**. Scale bar, 100 μm. The number of colonies (**B**) and EdU-positive cells (**D**) were counted in five fields. **E** Flow cytometry analysis of cell cycle distribution in these selected populations. And the percentage of three cell cycle phases was measured and compared among three groups (**F**). **G** Representative images of xenografic tumors inoculated subcutaneously of three clonal cells in NSG mice were shown. **H** Tumor weights were measured at the experimental end point (tumor volume exceeds 1000 mm3). **I** Tumor sizes of cell-derived xenografic bearing NTC, #1 and #18 cells were measured every 3 days. Data represent the mean ± SD of at least three independent experiments. **p < 0.01 and ***p < 0.001.

Given that cell cycle arrest is another typical feature of cellular senescence, we next examined the influence of ILK knock-out on the proliferative rate in MKN1 cells. By colony formation assay measuring anchorage-independent growth and EdU-incorporation assay assessing DNA synthesis rate in cells lacking ILK, we found significant growth inhibition in ILK deficient MKN1 and MKN28 cells (Fig. 2A–D, Supplementary Fig. S2A–D). Flow cytometry analysis showed a significant increase of G1 arrest in MKN1 KO#1 and #18 cells compared with NTC (Fig. 2E, F and Supplementary Fig. S10), whereas loss of ILK in MKN28 cells induced a robust G2 cell cycle arrest (Supplementary Fig. S2E, F, S10). Consistent with our in vitro data, the growth of xenografic tumor bearing ILK deficient MKN1 cells was significantly reduced in immune-compromised NOD-SCID-gamma (NSG) mice (Fig. 2G–I). Furthermore, immunohistochemistry analysis confirmed that the nuclear expression of PCNA, a proliferative marker, was markedly reduced in these xenografic tumor-bearing ILK KO MKN1 cells (Supplementary Fig. S2G, I). Apoptosis assays by flow cytometry on these selected populations showed a mild elevation in annexin V-stained apoptotic cells in MKN1 cells lacking ILK (Supplementary Fig. S2H, J). Taken together, these data above indicated that ILK loss induced G1 or G2 cell cycle arrest in GC cell lines.

### ILK deficient GC cells displayed an aberrant metabolic phenotype and mitochondrial dysfunction

**Fig. 3:**
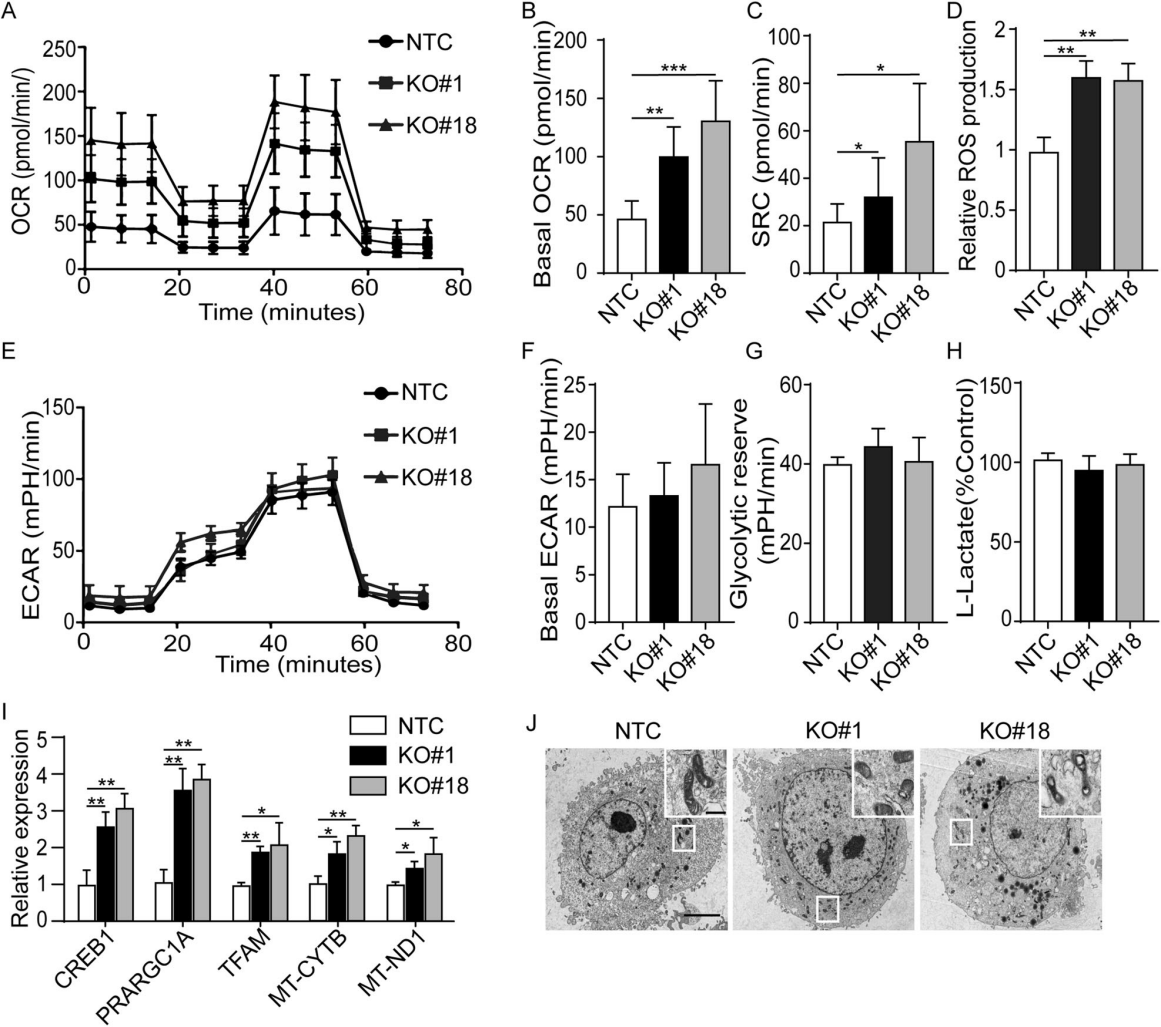
ILK KO cells displayed an aberrant metabolic phenotype and mitochondrial dysfunction in MKN1. **A** Oxygen consumption rate (OCR) was measured by the Seahorse analyzer in these clones with the treatment of oligomycin, FCCP and a mix of antimycin A and rotenone. **B** Basal OCR and **C** spare respiratory capacity (SRC) were determined as well. **D** Cellular ROS production was detected using a fluorescence microplate assay. **E** Extracellular acidification rate (ECAR) was measured using the Seahorse analyzer in these clones with the treatment of glucose, oligomycin and 2-deoxy-glucose (2-DG). **F** ECAR and **G** glycolysis reserve were determined from **E**. **H** Lactate production was measured in the culture medium of these cells. **I** qPCR analysis of metabolism including genes regulating OXPHOS and genes encoding mitochondrial proteins in MKN1 NTC, #1 and #18 clonal cells. **J** Representative images of transmission electron microscopy for observing the morphology and structure of mitochondria from these clones. Scale bar, 5 μm. Data represent the mean ± SD of at least three independent experiments. *p < 0.05, **p < 0.01, and ***p < 0.001.

Another typical phenotype of cells undergoing senescence is metabolic alteration [16]. We sought to investigate whether loss of ILK could result in metabolic reprogramming in these selected clones. KO clonal populations from MKN1 and MKN28 were subjected to assays for cell energy phenotyping. In cells from both lines, the basal oxygen consumption rate (OCR) and the OCR induced by the proton ionophore (uncoupler) FCCP (which measures maximal respiration capacity) were significantly increased in MKN1 and MKN28 cells lacking ILK expression compared to NTC controls (Fig. 3A, B, Supplementary Fig. S3A, B). Spare respiratory capacity (SRC), a measure of the ability of cells to respond to increased energy demands, exhibited an approximate 2 to 3-fold increase, suggesting the augmentation of respiratory activities in cells lacking ILK (Fig. 3C, Supplementary Fig. S3C). In contrast, the basal ECAR, which quantifies acid production in cell culture media and thus represents the glycolytic rate, was either unchanged in MKN1 ILK KO clones or significantly upregulated in MKN28 ILK KO cells compared to NTC cells (Fig. 3E, F, Supplementary Fig. S3E, F). Furthermore, the glycolytic reserve was not changed significantly in ILK KO MKN1 cells, while both MKN28 KO#17 and #18 cells had significantly increased glycolytic reserve with different magnitude. In line with these energetic phenotypes, increased ROS production was confirmed by luminescence sensitive ROS assay in all ILK KO clonal cells from two GC lines, whereas either no difference or moderate increase of lactate production was observed in selected MKN1 clonal lines or MKN28 ILK KO clones respectively (Fig. 3D, H, Supplementary Fig. S3D, H). Quantitative real-time PCR confirmed the increased expression of genes in the control of mitochondrial activity, such as *CREB1, PRARGC1A, TFAM, MT-CYTB* and *MT-ND1* in selected ILK KO clones from both lines (Fig. 3I, Supplementary Fig. S3I). Besides, unlike MKN28 derived clones with ILK depletion (Supplementary Fig. S3J), the major regulators of glucose metabolism such as *HIF1A, PKM2, LDHA HK2* and *PFKFB3* exhibited no alterations in MKN1 KO clones compared to NTC cells (data no shown). In addition, the pool of mitochondria in cells with ILK depletion had an abnormal appearance characterized by round, crista collapse, and vacuolization, which was associated with their aberrant energy phenotypes (Fig. 3J, Supplementary Fig. S3K). Given the phenotypic heterogeneity induced by ILK loss in MKN1 and MKN28, we examined another commonly used GC line, AGS. The depletion of ILK protein in AGS cells also led to metabolic reprogramming (increased OCR and ECAR), with the alteration of associated gene expression (Supplementary Fig. S9F–O). These observations were in the line with typical senescent phenotypes in AGS, such as increased β-gal activity, p21 and p53 expression (Supplementary Fig. S9A–E), and G1/G0 cell cycle arrest (Supplementary Fig. S11). Thus, the loss of ILK caused significant but distinct metabolic phenotypes in GC lines.

### ILK KO resulted in aberrant integrin signaling in GC

**Fig. 4:**
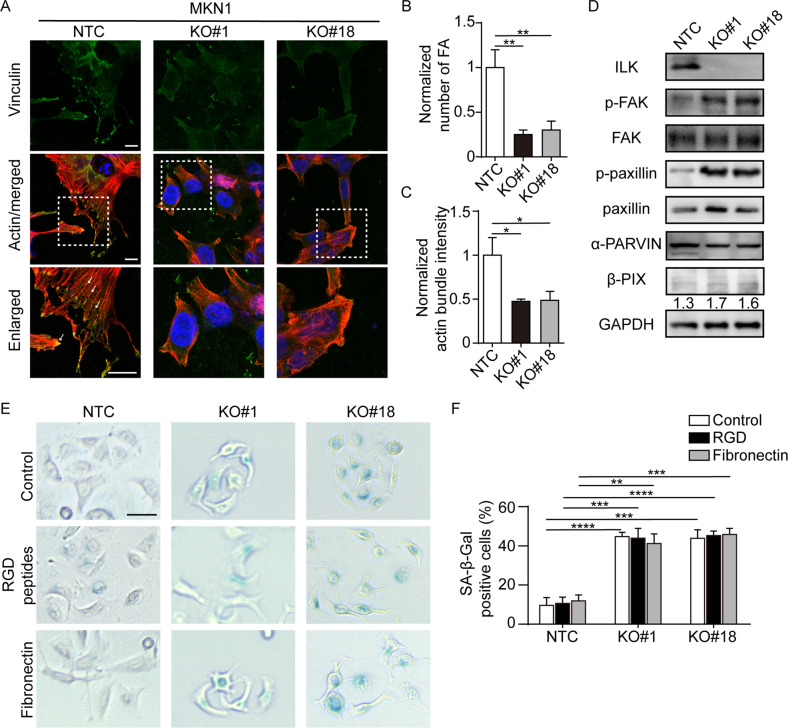
ILK depletion resulted in aberrant integrin signaling in MKN1. **A** Representative images of FAs (Vinculin positive) and actin bundles (red) were shown in MKN1 NTC and two ILK-KO clones. Arrows, vinculin-positive zone. Scale bar, 10 μm. **B** Quantification of the number of FAs. *n* ≥ 50 cells per group from three independent experiments. **C** Quantification of the intensity of actin bundles. *n* ≥ 50 cells per group from three independent experiments. **D** Cell lysates of three MKN1 clones were immunoblotted for a series of proteins related to integrin signaling. **E** Representative images for SA-β-Gal staining. NTC, #1 and #18 were incubated with control, RGD peptides and fibronectin for 5 days. Scale bar, 50 μm. **F** The percentage of cells with positive staining for SA-β-Gal from **E**. Data represent the mean ± SD of at least three independent experiments. *p < 0.05, **p < 0.01, ***p < 0.001, ****p < 0.0001.

As integrin signaling plays important role in cellular senescence, while ILK has been well identified as a key regulator of FA assembly and cytoskeleton dynamics, we next examined whether ILK loss mediated senescence is dependent on aberrant integrin signaling in these GC cells. We first investigated FA dynamics by immunofluorescence staining as senescence has often been closely linked with increased adhesive property, enlarged focal adhesion with denser actin stress fibers [17]. Unlike the phenotypes in senescent cells previously reported, loss of ILK in MKN1 and MKN28 significantly reduced FA formation with diminished F-actin bundling (Fig. 4A–C, Supplementary Fig. S4A–C). Furthermore, western blot results showed that ILK KO clones of both MKN1 and MKN28 displayed augmented phosphorylation of FAK at tyrosine 397 and strong induction of hyperphosphorylated paxillin at tyrosine 118 (Fig. 4D, Supplementary Fig. S4D). Notably, ILK depletion in both lines had mild impact on the expression of β-PIX (Fig. 4D, Supplementary Fig. S4D), which has been previously reported that the reduction of β-PIX during aging was associated with integrin-mediated senescence in human fibroblasts [17]. To determine whether enhanced or further destabilized FAs contribute to cellular senescence, we treated ILK depleted cells with RGD peptides (arginyl glycyl aspartic acid) to antagonize integrins and optimized the conditions of cell attachment by plating cells onto fibronectin precoated slides. As a result, neither RGD treatment nor precoating slides affected SA-β-gal activity in selected clonal cells from both lines (Fig. 4E, F, Supplementary Fig. S4E, F). Collectively, these data indicated that ILK depletion-induced senescence is not due to altered integrin signaling and is independent of β-PIX.

### LK mediated CME in GC lines

**Fig. 5:**
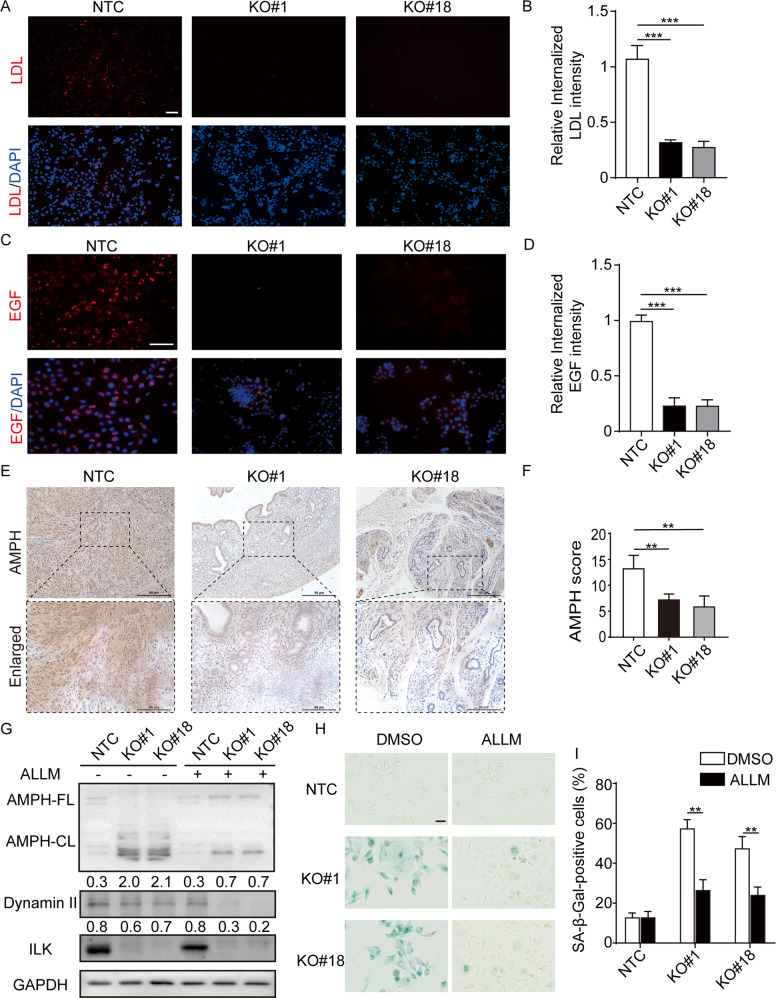
ILK deletion blocks CME resulting from amphiphysin I (AMPH) cleavage which induces senescence in MKN1. **A** Representative images of LDL endocytosis (red) were shown in MKN1 NTC and two ILK-KO clones. Scale bar, 100 μm. **B** Quantification of the number of LDL uptake. *n* ≥ 100 cells per group from three independent experiments. **C** Transferrin endocytosis (red) in MKN1 NTC and two ILK-KO clones. Scale bar, 50 μm. **D** Quantification of endocytosed transferrin. *n* ≥ 100 cells per group from three independent experiments. **E** Representative pairs of low power (up, solid line) and high power (low, dotted line) photomicrographs images of xenografic tumors that were subjected to AMPH staining were shown. Scale bar, 200 μm (solid line), 100 μm (dotted line). **F** The percentage of cells stained positively in the field for AMPH were shown by IHC scores. **G** The immunoblotting image showed AMPH cleavage by ILK knockout which can be reverse via calpain inhibition. The protein expression of AMPH-CL and Dynamin II were quantified by densitometry and normalization to GAPDH expression levels shown below the bands. **H** Effect of calpain inhibition on senescence. SA-β-Gal positive cells were quantified in **I**. *n* ≥ 100 cells per group from three independent experiments. Data represent the mean ± SD of at least three independent experiments. **p < 0.01 and ***p < 0.001.

As recent studies have identified the integrin signaling regulators contribute to the induction of cellular senescence via altered endocytosis [17, 18], we then measured receptor-dependent endocytosis in ILK depleted GC cells using fluorescent protein conjugated uptake assays. We found that loss of ILK significantly inhibited both LDL and EGF intracellular uptake (Fig. 5A–D, Supplementary Fig. 5A–D), indicating a general blockade of receptor-induced endocytosis in ILK-depleted cells. These results prompted us to further investigate whether ILK plays a role in AMPH (Amphiphysin) cleavage, which is a major endocytic adapter in CME and a known calpain substrate. We observed a strong reduction of AMPH staining in xenografic tumor tissue bearing MKN1 KO cells (Fig. 5E). Consistently, loss of ILK induced ~50% of full-length AMPH to a ~50 kDa N-terminal fragment (cleaved AMPH). The process of AMPH cleavage and ILK loss induced cellular senescence can be repressed by a calpain inhibitor, N-Acetyl-Leu-Leu-Methional (ALLM) (Fig. 5 G–I, Supplementary Fig. 5E–G). Intriguingly, there was also a decrease of dynamin II at greater level in KO#1 and KO#18 MKN1 cells (Fig. 5G, Supplementary Fig. 5E) in the presence of ALLM, suggesting that ILK may contribute to the protein stability of this molecule. Therefore, ILK loss induced cellular senescence via the perturbation of CME.

### ILK KO-induced senescence exhibited inflammation-associated transcriptomic patterns in GC cells

**Fig. 6:**
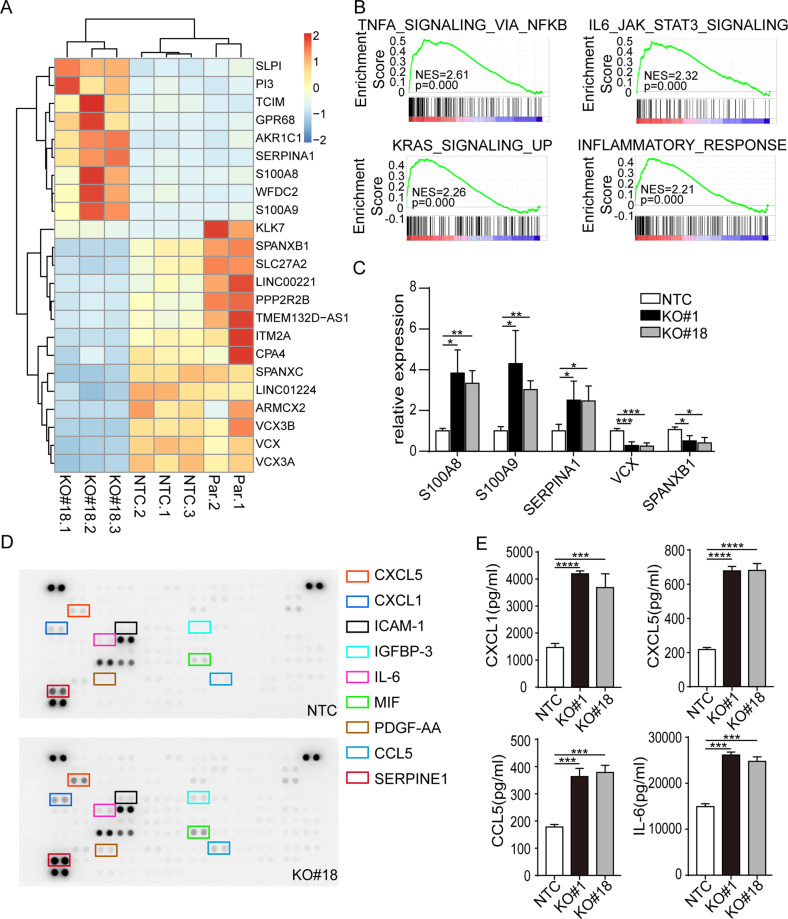
ILK loss-induced senescence exhibited inflammation-associated transcriptomic patterns in MKN1. **A** Heatmap displaying the most significantly differentially changed genes at RNA expression levels analyzed by RNA sequencing for NTC, ILK-KO #18 and parental MKN1 cells (adjusted p value <0.05, absolute logFC>1). **B** Gene set enrichment analysis (GSEA) identifying distinct pathways and biological processes between MKN1 NTC and #18 based on hallmark gene sets (h.all.v5.1.symbols.gmt). NES, normalized enrichment score. The top 4 enriched hallmark gene sets are shown as an example of maximum possible enrichment. **C** The alteration of selected genes expression was validated in NTC, #1 and #18 clones by qPCR analysis. **D** Cytokine antibody array was performed in the culture medium of MKN1 NTC and #18 according to the protocol from the manufacturer. Significantly changed cytokines in the absence of ILK ablation were shown on the right. Four selected cytokines were validated by ELISA **E**. Data represent the mean ± SD of at least three independent experiments. *p < 0.05, **p < 0.01, ***p < 0.001, ****p < 0.0001.

As senescence-associated gene expression and secretion are highly heterogeneous upon different senescence models [19], we sought to interrogate the steady-state transcriptomes of senescent cells by RNA-Seq. As shown in Fig. 5A, many immune-related genes such as *TCIM* (Transcriptional and Immune Response Regulator) involved in NF-κB signaling, and *S100 Calcium-binding proteins*, which play prominent roles in the regulation of immune responses and oxidant scavenging, were significantly elevated in MKN1 #18 cells. Interestingly, genes involved in male germ cell lineage, such as *SPANXB1*, *SPANXC*, *VCX/Y* family members, were downregulated (Fig. 6A). The expression of these selected genes that may play important roles in governing cellular senescence was further validated by qPCR (Fig. 6C). Furthermore, gene-set enrichment analysis comparing MKN1 #18 with NTC or parental control cells demonstrated that TNFA, STAT3, Inflammatory response and KRAS pathways were significantly enriched in the absence of ILK (Fig. 6B). While not as consistent on a molecule-by-molecule basis, inflammation- associated signaling pathways were also enriched in MKN28 cells lacking ILK expression, including STAT5, inflammatory response, and interferon signaling (Supplementary Fig. S6A, B).

The senescence-associated secretory phenotype (SASP) is another key feature of senescent cells. We speculated that the senescent MKN1 #18 cells may maintain the senescent state by secreting the aging-related proteins. We, therefore, measured the secretion of known SASP components in the cultured medium of ILK deficient cells compared to control cells using cytokine array. It revealed significantly increased secretion (fold change>2) of chemokines such as CXCL1, CXCL5, CCL5, and IL-6 in MKN1 ILK KO cells (Fig. 6D). Furthermore, other SASP ligands, such as IGFBP3 and SERPINE1 that were exclusively secreted in senescent epithelial cells but not in fibroblasts as previously described, were also elevated in the absence of ILK [19] (Fig. 6D). ELISA confirmed that these SASP factors were also significantly altered in another MKN1 clonal line (Fig. 6E). However, MKN28 with ILK depletion displayed distinct SASP. For example, GDF-15, which has recently been identified as one of the top “core” SASPs (Supplementary Fig. S6C) [19], was highly abundant and markedly elevated in MKN28 #18 clonal cells. DKK-1, the Wnt antagonist [20], and IL-8, the NF-κB activator [21], which have been reported significantly increased in senescent cells from different origins in response to various stimuli [22, 23], were also found to be upregulated in senescent MKN28 cells (Supplementary Fig. S6C, D). Altogether, despite the pro-inflammatory gene profiling pattern, heterogeneity of protein secretory phenotype was identified in ILK KO clonal populations from these two GC lines.

### The kinase activity of ILK is required for the regulation of cellular senescence

**Fig. 7:**
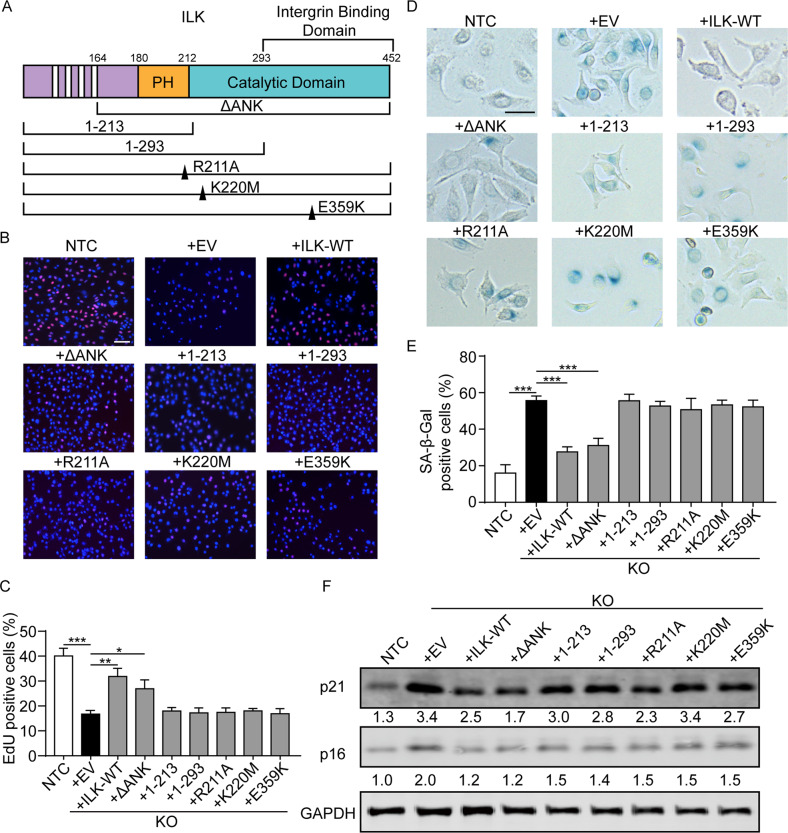
The kinase activity of ILK is required for the regulation of cellular senescence. **A** V2-tagged ILK variants were transfected into MKN1 cells. They are ILK constructs lacking the four ankyrin repeats (ΔANK), the catalytic domain (Δ1–213), integrin binding region (Δ1–293), as well as the dominant negative ILK mutant (R211A), ILK with disrupted catalytic function (K220M) and a mutant deficient for β-parvin binding (E359K). **B** Representative images of EdU assay were shown with the MKN1 NTC and ILK-KO transfected with different ILK mutants and truncations. Scale bar, 100 μm. Quantification of EdU positive cells was displayed in **C**. **D** Representative images of SA-β-Gal staining of MKN1 NTC and ILK-KO transfected with empty, different ILK mutants and truncations. Scale bar, 50 μm. Quantification of the percentage of cells with positive staining for SA-β-Gal was displayed in **E**. **F** Cell lysates were analyzed by western blotting using antibodies to p21 and p16 with GAPDH as the loading control. Data represent the mean ± SD of at least three independent experiments. *p < 0.05, **p < 0.01, ***p < 0.001.

Next, we sought to localize the functional domain of ILK that mediates cellular senescence by performing EdU assays using tagged truncations and function-disrupting ILK point mutants. Truncated EGFP-ILK variants containing the ankyrin repeat and pleckstrin homology-like domains (amino acids 1–213 and 1–293) did not rescue ILK loss induced cellular senescence. In contrast, an N-terminal truncated ILK variant (ΔANK) containing the pleckstrin homology-like and catalytic domains reversed proliferative inhibition to equally as well as WT-ILK (Fig. 7A–C, Supplementary Fig. 7A, B). ILK-K220M, a point mutation to inhibit ILK catalytic activity, failed to reverse senescence in ILK-depleted MKN1 and MKN28 cells. This negative observation was also shown in cells transfected with ILK-E359K, which is unable to bind to β-parvin, and ILK-R211A, which acts as a dominant-negative ILK signaling mutant (Fig. 7A–C, Supplementary Fig. 7A, B). These results indicated that the catalytic domain of ILK mediates cellular senescence in MKN1 cells. Consistently, overexpression of ILK lacking the N-terminus- ANK repeats or the full-length ILK in senescent ILK KO cells were both capable of restraining not only β-Gal activity induced by ILK KO but also the elevation of p21 and p16 expression (Fig. 7D–F, Supplementary Fig. 7C–E). Interestingly, despite no alteration in proliferation, overexpression of ILK E359K mutant partially reversed the number of β-Gal positive cells and expression of p16 in the senescent MKN1 and MKN28 cells.

**Fig. 8:**
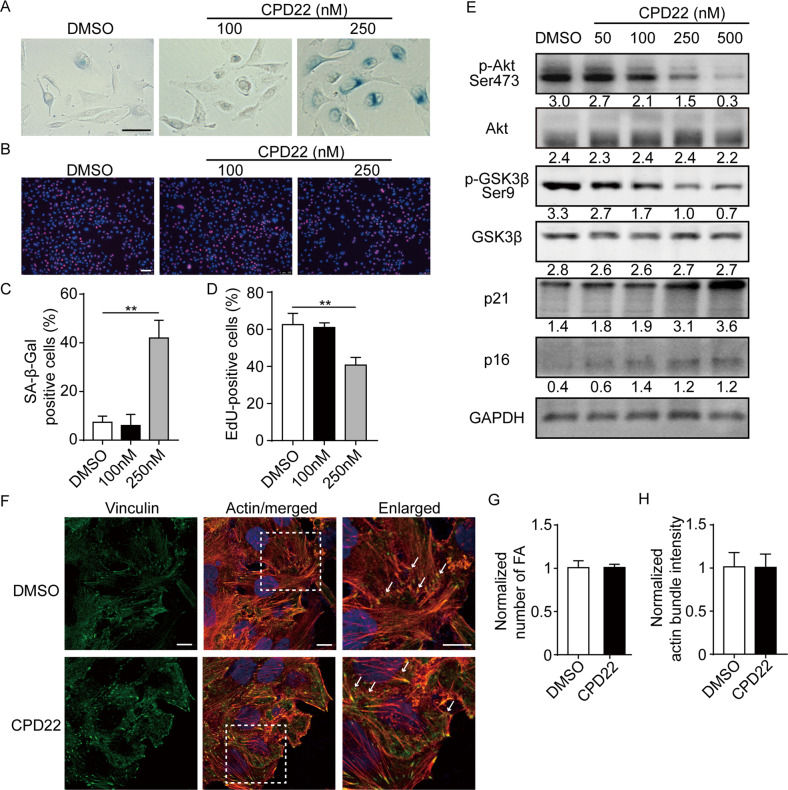
ILK-mediated cellular senescence was associated with its kinase function in MKN1. **A** Representative images of SA-β-Gal staining were shown with various concentrations of the ILK kinase inhibitor, Cpd22 for 5 days. Scale bar, 50 μm. Quantification of positive cells for the SA-β-Gal assay in A was displayed in **B**. **C** Representative images of EdU assay were shown as the dosage of Cpd22 treatment indicated. Scale bar, 100 μm. Quantification of EdU positive staining in C was displayed in **D**. **E** MKN1 was treated with Cpd22 as indicated for 5 days. Cell lysates were then harvested and immunoblotted for p-Akt (Ser473), p-GSK3β (Ser9), p21 and p16, with GAPDH as the loading control. **F** Representative images of FAs (Vinculin stained) and actin bundles (red) in MKN1 cells incubated with DMSO vehicle or 250 nM Cpd22 for 5 days. Arrows, vinculin-positive zone. Scale bar, 10 μm. Quantification of number of **G** FAs and **H** intensity of actin bundles. *n* ≥ 50 cells per group from three independent experiments. Data represent the mean ± SD of at least three independent experiments. **p < 0.01.

In light of observed results of those ILK mutants, we then tested the effects of the ILK specific inhibitor, CPD22, in cellular senescence, which was known to have in vitro anti-proliferative potency against a few human cancer cells at IC50 (2–5 μM) [24] and we tested the IC50 in MKN1 and MKN28 cell lines (Supplementary Fig. S8A). By serial dilution, we found that using 5-day treatment of CPD22, the compound inhibited ILK kinase activity at the dose of 250 nM, and increased senescent cells in MKN1, which coincided with the downregulation of p21 and p16, as well as the reduction of EdU incorporated cells (Fig. 8A–E). We also got similar findings in MKN28 (Supplementary Fig. S8B–F). Notably, the compound inhibiting ILK activity at this concentration did not lead to the disruption of focal adhesion and cytoskeleton dynamics (Fig. 8F–H, Supplementary S8G–I). In contrast, two downstream effectors, AKT and GSK3β were significantly hypo-phosphorylated in CPD22 treated cells compared to untreated control (Fig. 8E, Supplementary Fig. S8F), in keeping with the inhibitory effects on cell growth in the presence of ILK inhibitor in other cancer lines as previously described [2, 5]. Collectively, these results indicated that ILK kinase activity is required for its regulation of cellular senescence.

## Discussion

Despite the broad cellular functions in the physiology and pathology of ILK, the role of ILK in cellular senescence has not been elucidated. A previous study showed that PPARβ/δ promotes HRAS-induced senescence and skin tumor suppression by repressing p-AKT signalling via ILK repression [25]. In another study, anti-ILK therapy using small molecule inhibitor QLT0267 induces cellular senescence of retinoblastoma cells in an Rb-dependent manner [15]. Our study first demonstrated that ILK depletion by CRISPR/Cas9 in gastric cancerous epithelial lines, with different histology induced potent cellular senescence. In contrast to previous studies, senescent cells from both lines expressed higher levels of phosphorylated Rb, the inactive form of this cell cycle regulator, suggesting a Rb-independent mechanism for ILK loss associated cellular senescence and cell cycle arrest.

Cellular senescence is often characterized by a hyper-adhesive phenotype with enhanced FAs and actin stress fibers. A recent study has also reported that declining β-PIX protein level with age in mouse and human lung tissue coincided with the upregulation of various senescence markers, including increased expression of p16, phosphorylated nuclear p-γH2AX and SA-β-Gal activity. β-PIX regulates cellular senescence via its suppression of CME through direct competition of GIT1/2 for the calpain-binding site on paxillin [17]. Similarly, loss of ILK also induced calpain cleavage of the endocytic adapter AMPH to suppress CME. ILK and Calpain might compete for binding to AMPH during FA dynamics. Loss of ILK, in turn, increased the association of calpain proteases and AMPH, leading to calpain-mediated proteolysis of AMPH. Interestingly, β-PIX was slightly increased in ILK-depleted GC cells, reflecting a dispensable role of β-PIX in this effect. Thus, ILK might alternatively interacts with another focal adhesion adapter protein, paxillin, which is also a calpain substrate and capable of sensing oxidative stress [26]. In this study, we found that loss of ILK significantly increased tyrosine 118 phosphorylation of paxillin, a key regulator of both assembly and the turnover of adhesion complexes. As paxillin Tyr 118 localized in between the leucine-rich LD 1 and LD2 domains, the motifs are preferentially recognized by calpain proteases [27]. Aberrant paxillin signaling induced by ILK depletion may confer deregulation of calpain-dependent proteolysis as the consequences of a few cancer-associated-somatic mutations in the N-terminal of paxillin previously described [26].

By employing various ILK mutants and specific ILK inhibitors, we next confirmed that selective inhibition of ILK kinase but not affecting its binding partner is responsible for the induction of senescent state in these cancerous cells. The senescent state of MKN1 and MKN28 induced by CPD22 did not affect FA dynamics. Indeed, the dose (at nM level) used in our study was much lower than that in previous studies, in line with the notion of the preferential dependency of ILK functions in cancer cell survival regardless of adhesion-dependent or independent manner than integrin-mediated cell attachment [28].

Another interesting finding is heterogeneous phenotypes in GC cells during senescence. For example, senescent MKN1 and AGS cells displayed increased G1 phase arrest while ILK null MKN28 cells showed cessation of the cell cycle in the G2 phase. Considering similar findings of disparity between cancerous and non-cancerous epithelial cells in the regulation of cell cycle and related protein expression, it may be due to different dependencies on ILK activity in the context of the cell cycle [2, 11, 29]. Furthermore, the heterogeneity of ILK-dependent senescence was also observed in energetic assays. Although clones from both lines exhibited augmented oxygen consumption and increased mitochondrial activities, MKN28 and AGS but not MKN1 had significantly enhanced glycolysis activity. Whether these metabolic differences in the absence of ILK may confer metastatic potential, energy sources, and intrinsic metabolic traits [30] needs further studies.

SASP is another feature of cellular senescence which have been extensively studied in the context of different cell origin and stimuli, but the heterogeneity of SASP composition limits its use as a specific senescence marker [16, 19]. Except for a few typical senescence- associated secretory factors previously reported, such as IL-6, IGFBPs, and CXCLs, we detected exclusive secretory proteins in cultured media of senescent MKN1 and MKN28 cells, respectively. For example, GDF-15, a significantly increased protein in MKN28 clones, has recently been identified as an aging marker in human serum. It has also been reported that GDF-15 plasma level is associated with the severity of side effects in cancer patients following platinum/ cisplatin-based chemotherapy [31]. As many genotoxic chemotherapies are known to induce cellular senescence in normal cells [32], these studies suggest inhibition of ILK activity can be used as a more specific senescence inducer in cancer cells, as the survival and growth of these cancerous cells are addicted to activated ILK function. In addition, loss of ILK in gastric cancerous cells appeared to induce cell-intrinsic pro-inflammatory signaling pathways. However, heterogeneity of these secreted cytokines and chemokines may facilitate to shape a complex tumor microenvironment, influencing the efficacy of anti-tumor treatment in clinical settings.

In summary, our study demonstrated a non-canonical role of ILK regulating cellular senescence in GC, characterized by the inhibition of the cell cycle, increased lysosomal content, and aberrant metabolic phenotypes. We provide a new insight into the connection of ILK-senescence- inflammatory signaling pathway could be a new target for effective cancer therapy for GC.

## Supplementary information

supplementary materials

Western blot Original Data File

AJ checklist

## Data Availability

The RNA sequencing data of MKN1 and MKN28 for parental, NTC and ILK KO clone population has been deposited as well as the data of cytokine array in figshare. 10.6084/m9.figshare.14386520.v1
