# Supplementary material for: The kinase activity of integrin-linked kinase regulates cellular senescence in gastric cancer
Source: Cell Death Dis. 2022 Jul 1;13(7):577. doi: 10.1038/s41419-022-05020-3 (PMC9249761; doi:10.1038/s41419-022-05020-3)
Supplement: Supplementary file 1 — supplementary materials [file 41419_2022_5020_MOESM1_ESM.docx]

**Supplementary materials**

**The kinase activity of integrin linked kinase regulates cellular senescence in gastric** **cancer**

Chengbo Ji, Mili Zhang, Junjie Hu, Can Cao, Qisheng Gu, Youdong Liu, Xu Li, Duogang Xu, Hugh Gao, Le Ying, Yuqin Yang, Jikun Li, Liang Yu

**Supplementary Figure S1.** Deletion of ILK in MKN28 induced cellular senescence.

**Supplementary Figure S2.** MKN28 lacking ILK displayed significant G2 cell cycle arrest.

**Supplementary Figure S3.** ILK KO displayed an aberrant metabolic phenotype and mitochondrial dysfunction in MKN28.

**Supplementary Figure S4.** ILK depletion resulted in aberrant integrin signaling in MKN28. **Supplementary Figure S5.** ILK deletion inhibited CME in MKN28.

**Supplementary Figure S6.** ILK loss induced senescence exhibited inflammation-associated transcriptomic pattern in MKN28.

**Supplementary Figure S7.** The kinase activity of ILK is required for the regulation of cellular senescence.

**Supplementary Figure S8.** ILK mediated cellular senescence was associated with its kinase function in MKN28.

**Supplementary Figure S9.** ILK KO induced cellular senescence and metabolic dysfunction in AGS.

**Supplementary Figure S10.** Flow cytometry analysis of cell cycle distribution in the cell lines indicated.

**Supplementary Figure S11.** Flow cytometry analysis of cell cycle distribution in AGS clones.

**Supplementary Table S1.** Primer sequences used for SYBR qPCR gene expression assays.

**
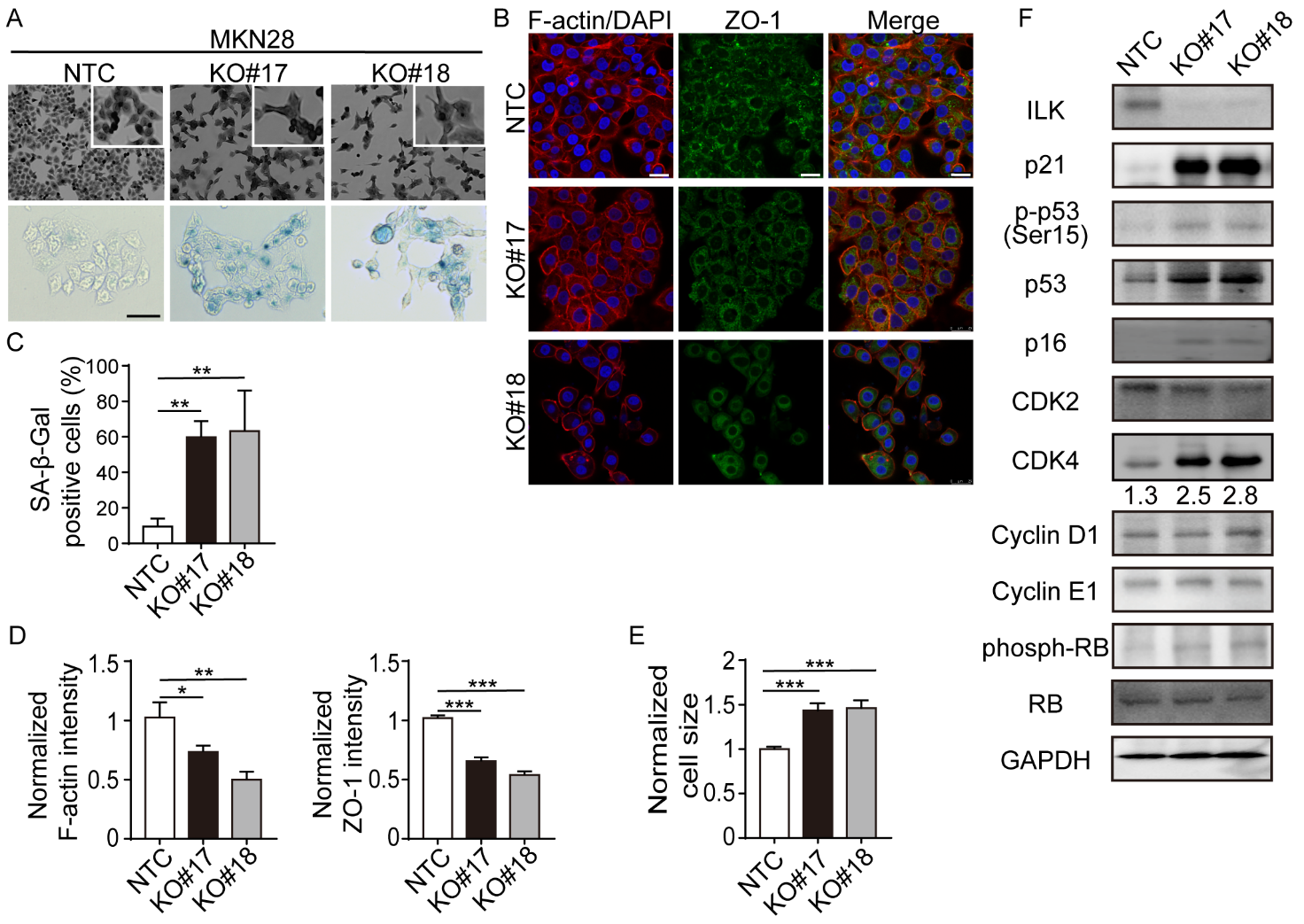
**

**Supplementary Figure S1. Deletion of ILK in MKN28 induced cellular senescence.**

(A) Crystal violent staining under microscopy of MKN28 NTC cells and ILK knockout clone #17 and #18 with SA-β-Gal staining respectively. Scale bar, 50μm. (B) F-actin (red)/DAPI (blue) and ZO-1 (green) staining of three selected clones. Scale bar, 25μm. (C) The percentage of cells with positive staining for SA-β-Gal from A. (D) Quantification of F-actin and ZO-1 fluorescent intensity from C. (E) Flow cytometry analysis of cell size from indicated clones. (F) Western blot analysis of cell cycle related proteins in the lysate of three clones, with GAPDH as the loading control. Data represent the mean ± SD of at least three independent experiments. *p < 0.05, **p < 0.01 and ***p < 0.001.


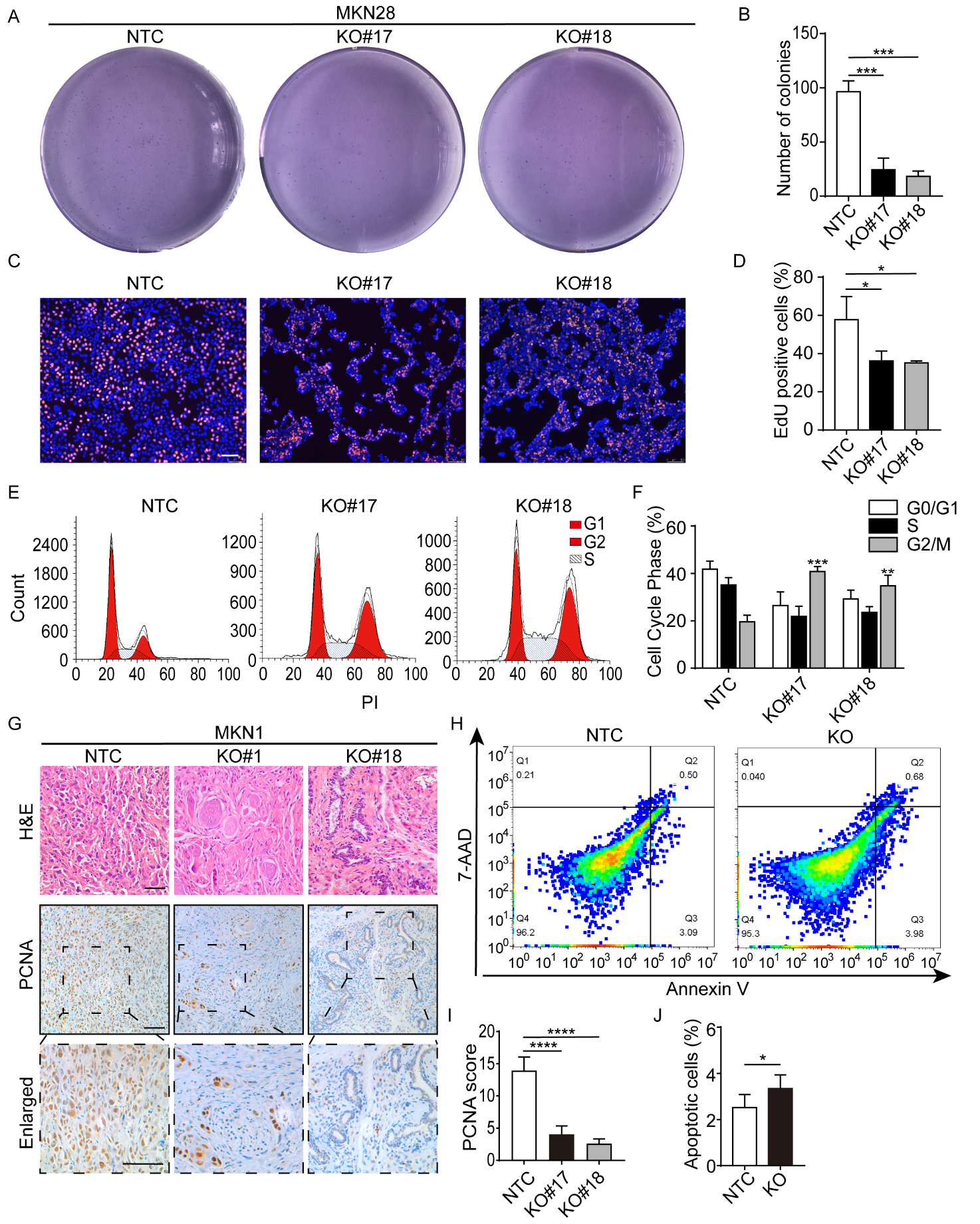


**Supplementary Figure S2. MKN28 lacking ILK displayed significant G2 cell cycle arrest.**

The proliferation of three MKN28 clones as indicated was measured by soft agar assays (A) and EdU (C). Scale bar, 100μm. Number of colonies (B) and EdU positive cells (D) were counted. (E) Flow cytometry analysis of cell cycle distribution in these selected populations. And the percentage of three cell cycle phases were measured and compared in three MKN28 clones (F). (G and I) Representative low power H&E stained, low power (left, solid line) and high power (right, dotted line) photomicrographs of PCNA-immunostaining MKN1 Xenografts. Scale bars, 200mm (low power) and 100mm (high power). (H and J) Flow cytometry analysis of apoptosis in MKN1 NTC, and ILK-KO cells. Data represent the mean ± SD of at least three independent experiments. *p < 0.05, **p < 0.01, ***p < 0.001 and ****p<0.0001.


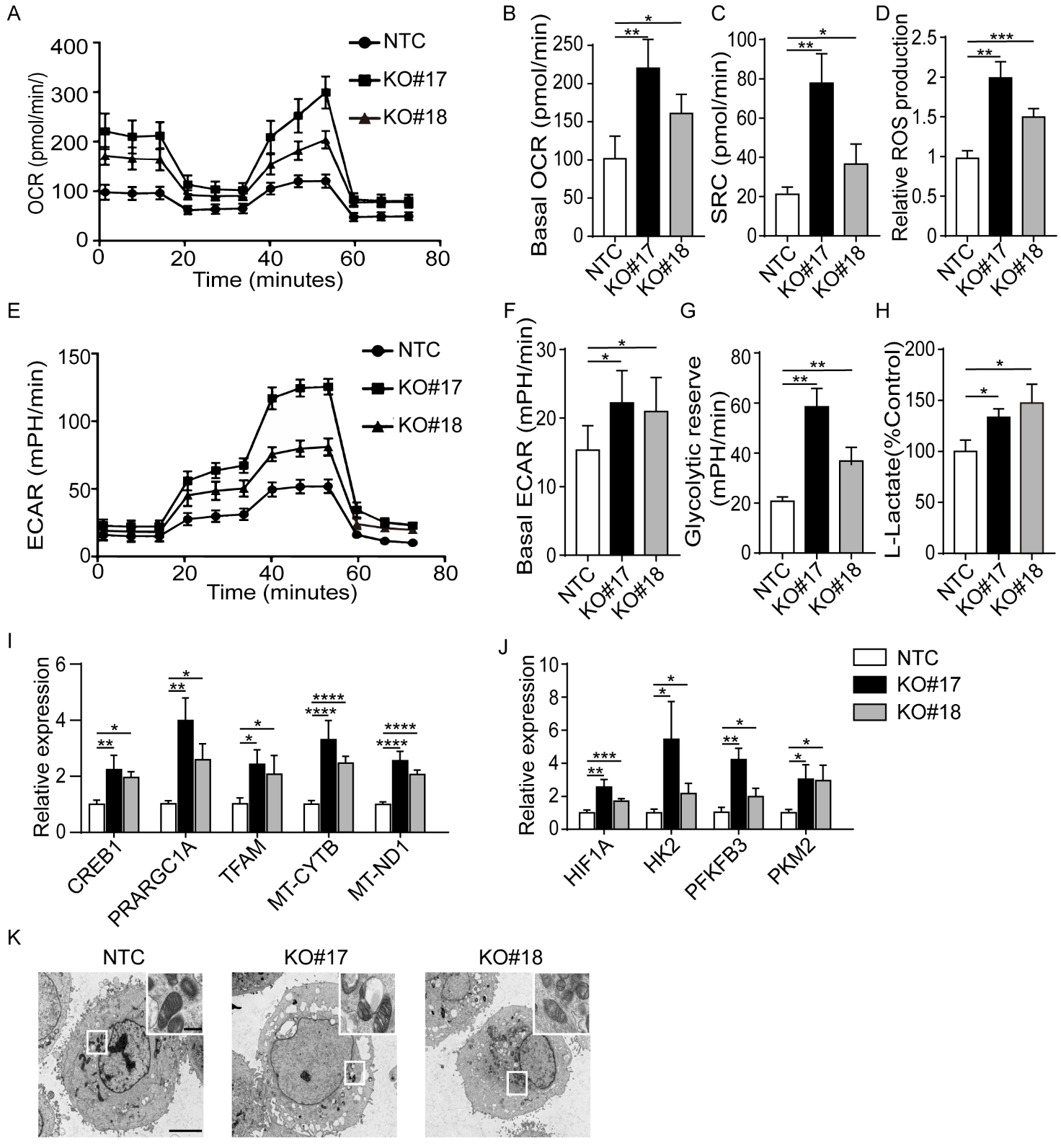


**Supplementary Figure S3. ILK KO displayed an aberrant metabolic phenotype and mitochondrial dysfunction in MKN28.**

(A) Oxygen consumption rate (OCR) was measured by the Seahorse analyzer in these clones with the treatment of oligomycin, FCCP and a mix of antimycin A and rotenone. (B) Basal OCR and (C) spare respiratory capacity (SRC) were determined as well. (D) Cellular ROS production was detected using a fluorescence microplate assay. (E) Extracellular acidification rate (ECAR) was measured using the Seahorse analyzer in these clones with the treatment of glucose, oligomycin and 2-deoxy-glucose (2-DG). (F) ECAR and (G) glycolysis reserve were determined from E. (H) Lactate production was measured in the culture medium of these cells. (I) qPCR analysis of metabolism including genes regulating OXPHOS and genes encoding mitochondrial proteins in MKN28 NTC, #17 and #18 clonal cells. (J) qPCR analysis of genes regulating glycolysis in three MKN28 clones. (K) Representative images of transmission electron microscopy for observing the morphology and structure of mitochondria from these clones. Scale bar, 5μm. Data represent the mean ± SD of at least three independent experiments. *p < 0.05, **p < 0.01, ***p < 0.001, ****p<0.0001.

**
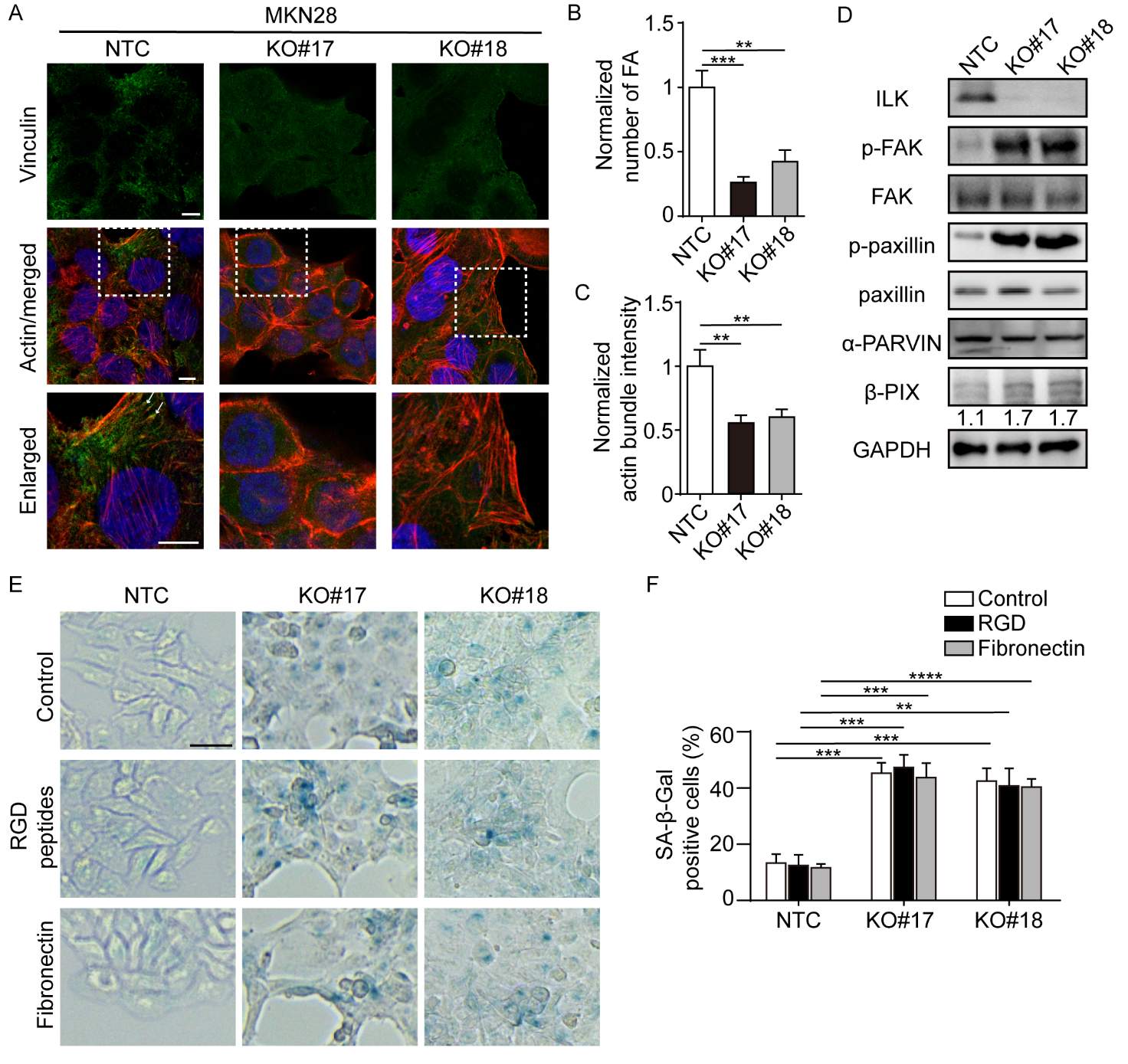
**

**Supplementary Figure S4. ILK depletion resulted in aberrant integrin signaling in MKN28.**

(A) Representative images of FAs and actin bundles (red) were shown in MKN28 NTC and two ILK-KO clones. Arrows, vinculin-positive zone. Scale bar, 10μm. (B) Quantification of number of FAs. n ≥ 50 cells per group from three independent experiments. (C) Quantification of intensity of actin bundles. n ≥ 50 cells per group from three independent experiments. (D) Cell lysates of three MKN28 clones were immunoblotted for a series of proteins related to integrin signaling. (E) Representative images for SA-β-Gal staining. NTC, #17 and #18 were incubated with control, RGD peptides and fibronectin for 5 days. Scale bar, 50μm. (F) The percentage of cells staining positive for SA-β-Gal from E. Data represent the mean ± SD of at least three independent experiments. **p < 0.01, ***p < 0.001, ****p<0.0001.

**
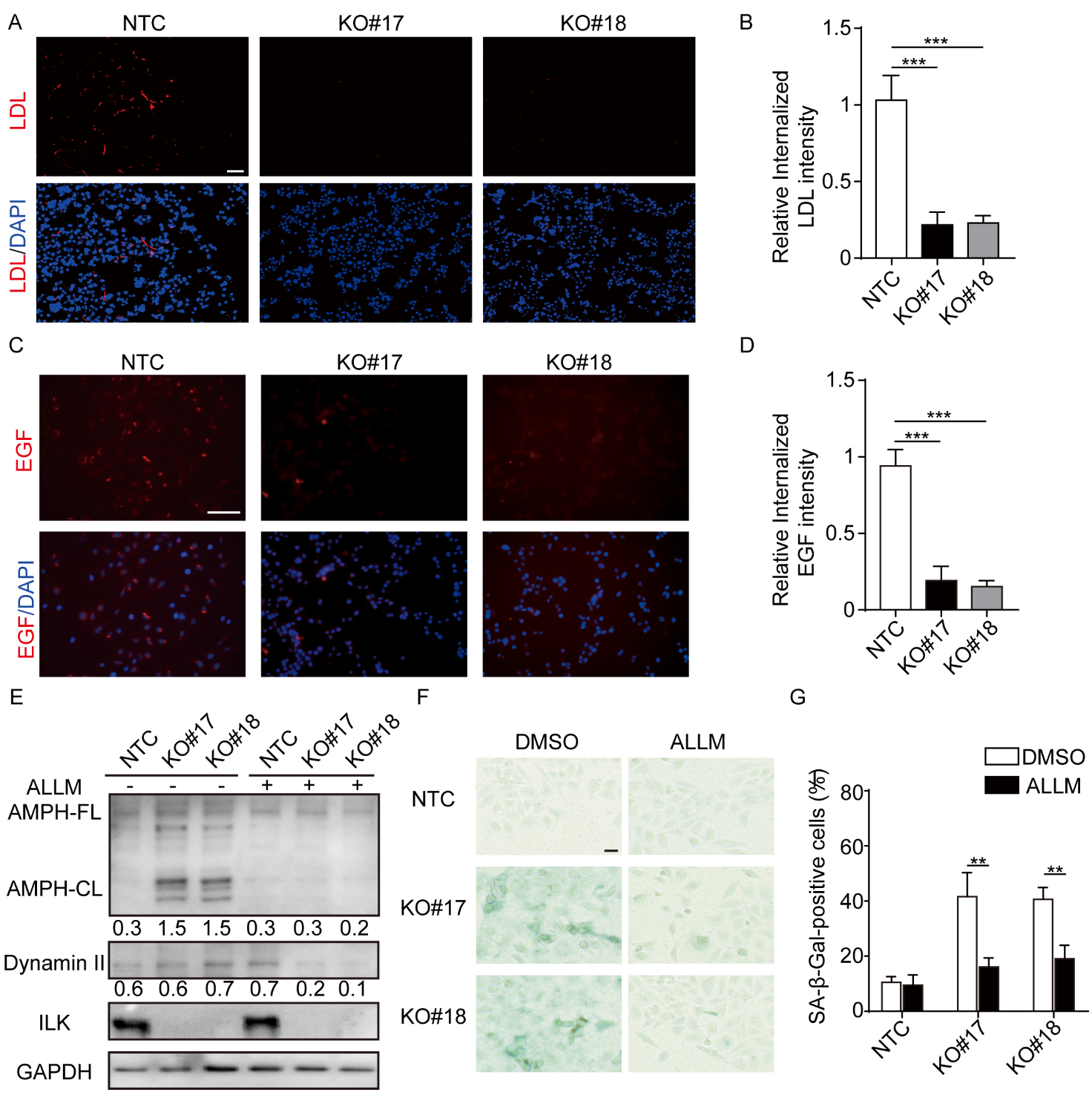
**

**Supplementary Figure S5. ILK deletion inhibited CME in MKN28.**

(A) Representative images of LDL endocytosis (red) were shown in MKN1 NTC and two ILK-KO clones. Scale bar, 100μm. (B) Quantification of number of LDL uptake. n ≥ 100 cells per group from three independent experiments. (C) Transferrin endocytosis (red) in MKN28 NTC and two ILK-KO clones. Scale bar, 50μm. (D) Quantification of endocytosed transferrin. n≥100 cells per group from three independent experiments. (E) Immunoblotting image showed AMPH cleavage by ILK knockout which can be reverse via calpain inhibition. The protein expression of AMPH-CL and Dynamin II were quantified by densitometry and normalization to GAPDH expression levels shown below the bands. (F) Effect of calpain inhibition on senescence. SA-β-Gal positive cells were quantified in (G). n≥100 cells per group from three independent experiments. Data represent the mean ± SD of at least three independent experiments. **p < 0.01 and ***p < 0.001.


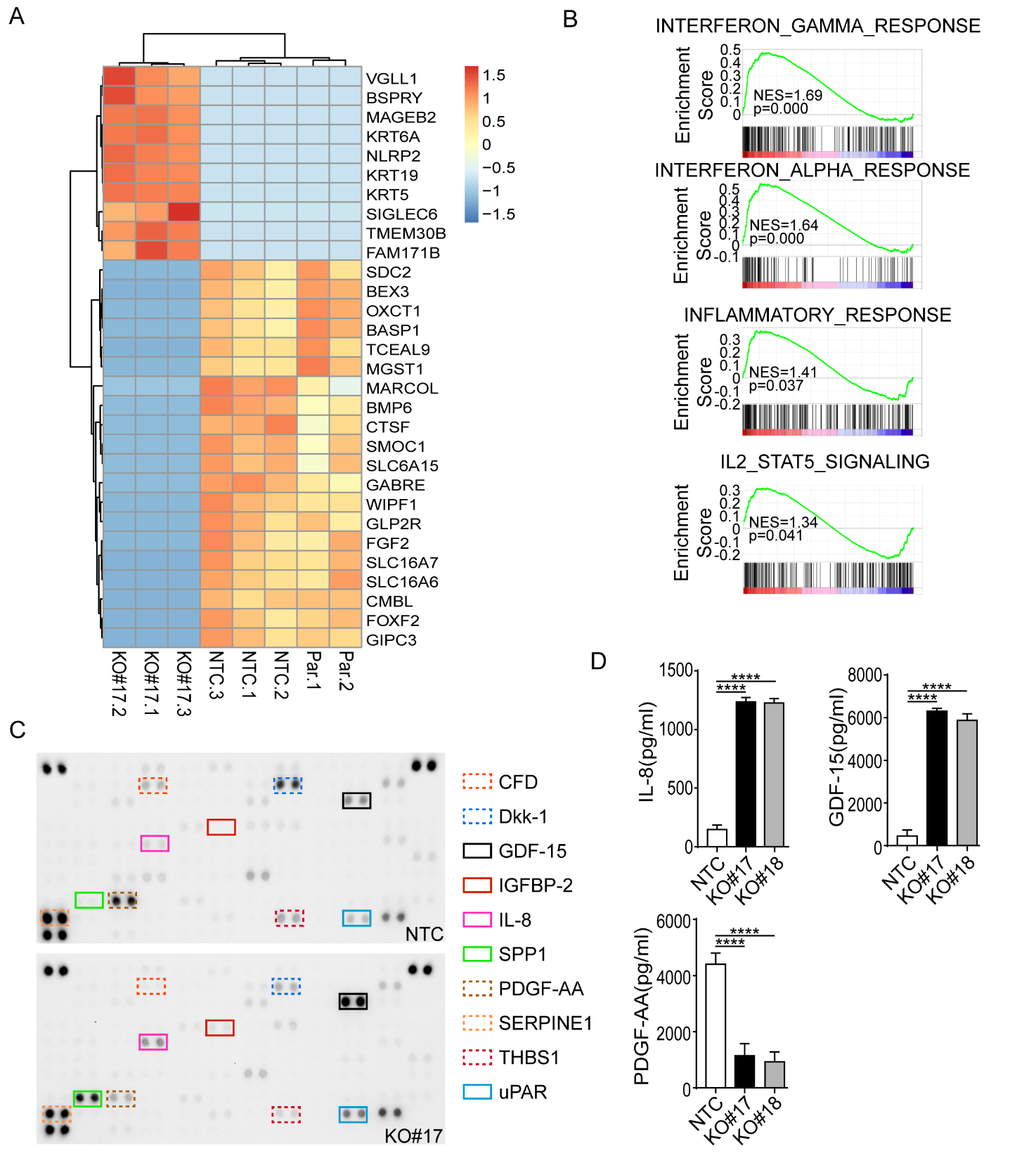


**Supplementary Figure S6. ILK loss induced senescence exhibited inflammation-associated transcriptomic pattern in MKN28.**

(A) Heatmap displaying the most significantly differentially changed genes at RNA expression levels analyzed by RNA sequencing for NTC, ILK-KO #17 and parental MKN28 cells (p adjusted. value <0.05, absolute logFC>1). (B) Top 4 enriched hallmark gene sets in the #17 cells compared with those of NTC cells identified by GSEA. (C) Cytokine antibody array was performed in culture medium of MKN28 NTC and #17 clones according to protocol provided by the manufacturer. Cytokines induced (solid line) or reduced (dotted line) with ILK ablation were shown on the right. Three selected cytokines were validated by ELISA (D). Data represent the mean ± SD of at least three independent experiments. ****p<0.0001.

**
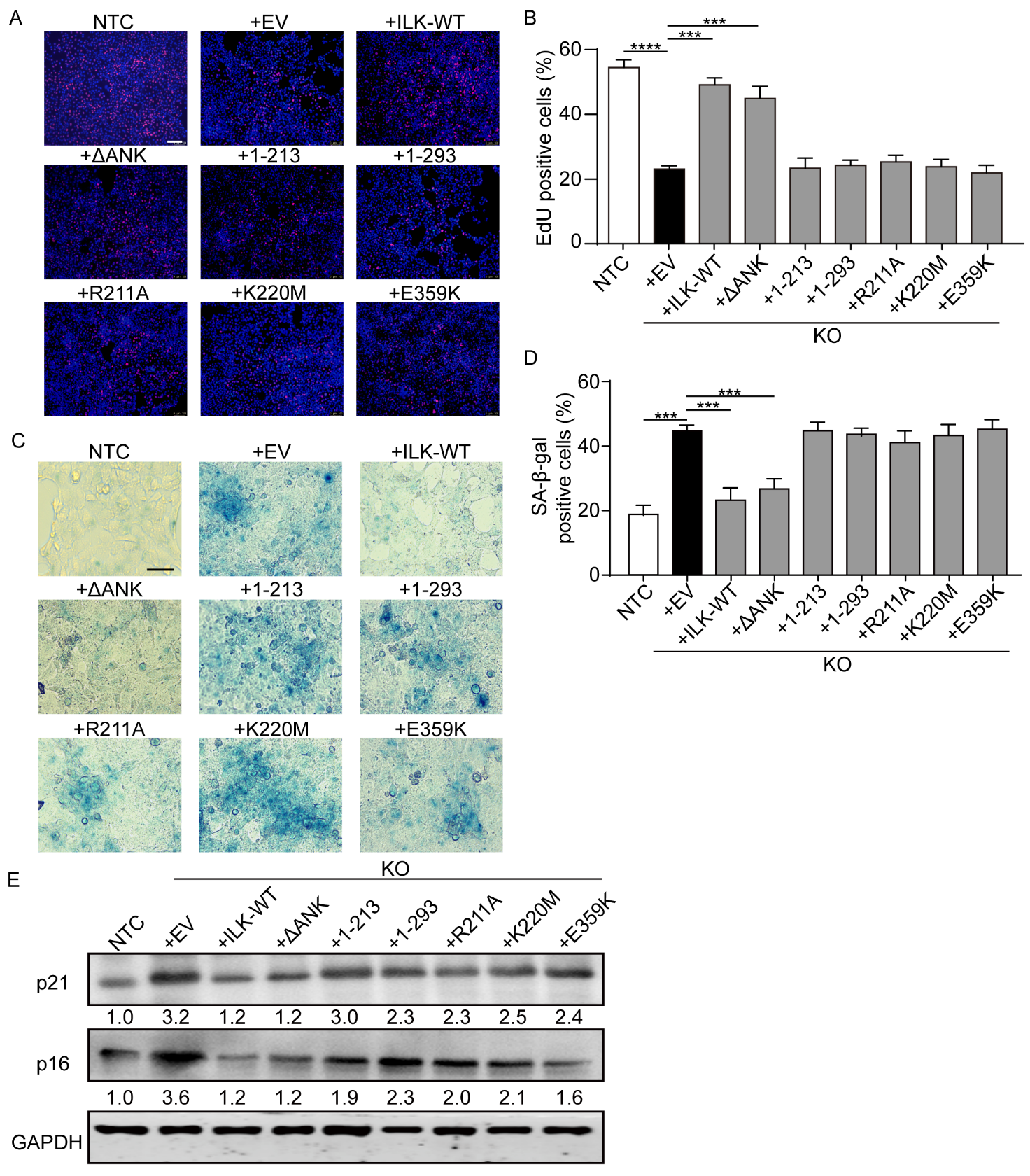
**

**Supplementary Figure S7. The kinase activity of ILK is required for the regulation of cellular senescence.**

(A) Representative images of EdU assay were shown with the MKN28 NTC and ILK-KO transfected with different ILK mutants and truncations. Scale bar, 100μm. Quantification of EdU positive cells was displayed in (B). (C) Representative images of SA-β-Gal staining of MKN28 NTC and ILK-KO transfected with empty, different ILK mutants and truncations. Scale bar, 50μm. Quantification of the percentage of cells with positive staining for SA-β-Gal were displayed in (D). (E) Cell lysates were analyzed by western blotting using antibodies to p21 and p16 with GAPDH as the loading control. Data represent the mean ± SD of at least three independent experiments. ***p < 0.001, ****p < 0.0001.

**
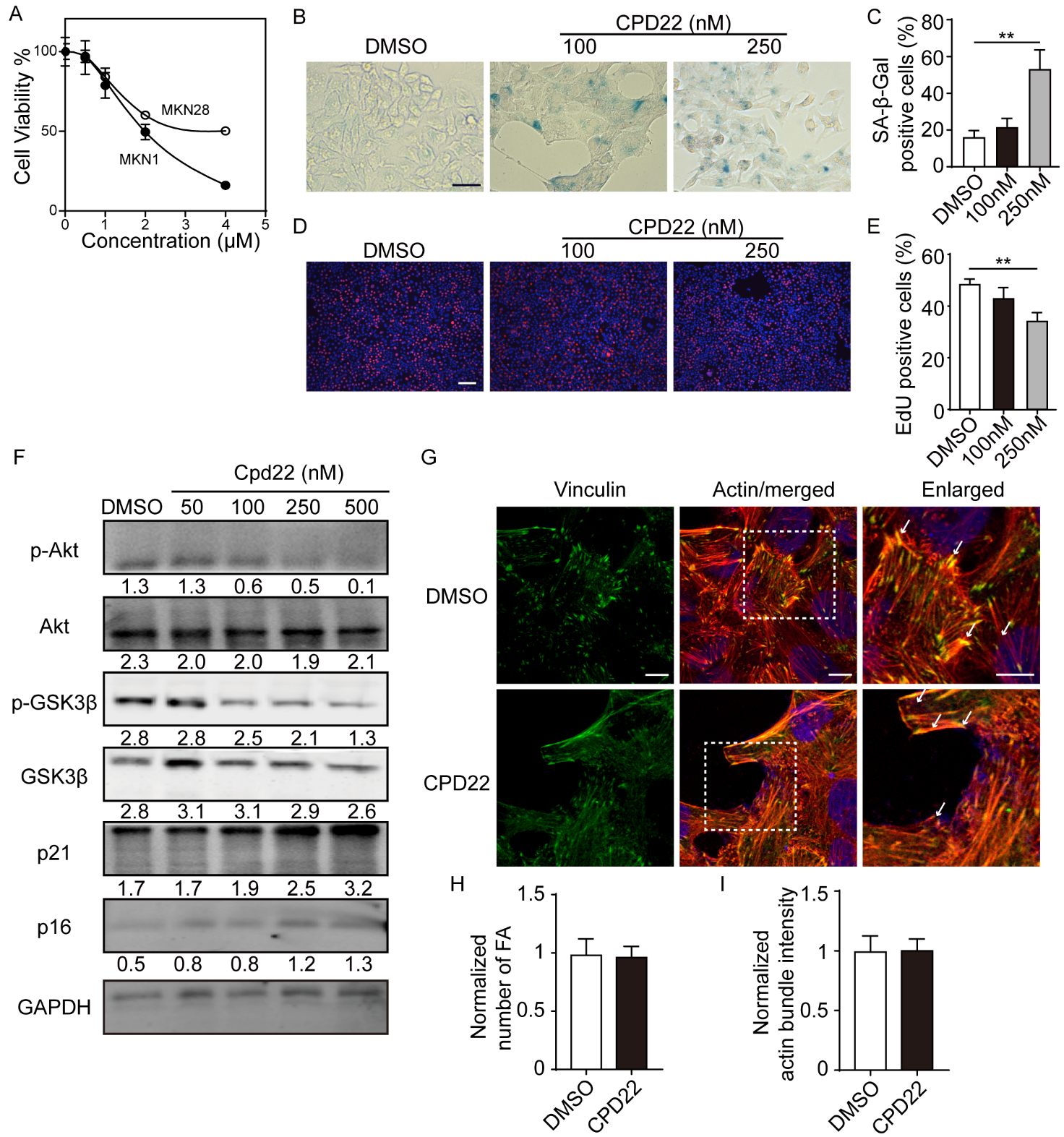
**

**Supplementary Figure S8. ILK mediated cellular senescence was associated with its kinase function in MKN28.**

(A) Dose-dependent suppressive effects of CPD22 on the viability of MKN1 and MKN28 after 24h of treatment. Cell viability was determined by CCK8 assays. Points, means; bars, SD (n = 6). (B) Representative images of SA-β-Gal staining were shown with various concentration of the ILK kinase inhibitor, Cpd22 for 5 days. Scale bar, 50μm. Quantification of positive cells for SA-β-Gal assay in B were displayed in (C). (D) Representative images of EdU assay were shown as the dosage of Cpd22 treatment indicated. Scale bar, 100μm. Quantification of EdU positive staining in D were displayed in (E). (F) MKN28 was treated with Cpd22 as indicated for 5 days. Cell lysates were then harvested and immunoblotted for p-Akt (Ser473), p-GSK3β (Ser9), p21 and p16, with GAPDH as the loading control. (G) Representative images of FAs (Vinculin stained) and actin bundles (red) in MKN28 cells incubated with DMSO vehicle or 250nM Cpd22 for 5 days. Arrows, vinculin-positive zone. Scale bar, 10μm. Quantification of number of FAs (H) and actin bundles intensity (I). n ≥ 50 cells per group from three independent experiments. Data represent the mean ± SD of at least three independent experiments. **p < 0.01.

**
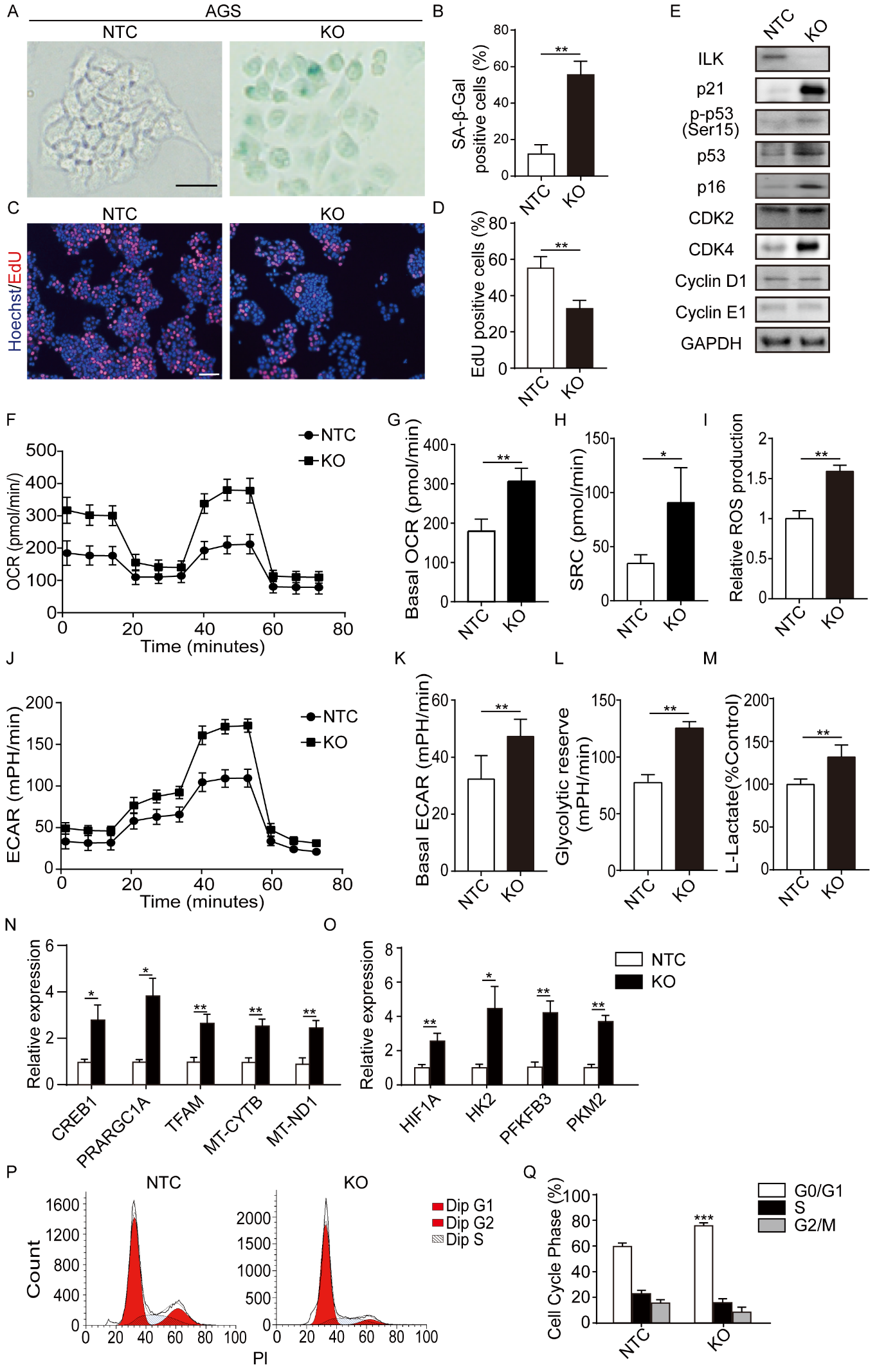
**

**Supplementary Figure S9. ILK KO induced cellular senescence and metabolic dysfunction in AGS.**

(A) NTC and ILK knock-out of AGS with SA-β-Gal staining respectively. Scale bar, 50μm. (B) The percentage of cells with positive staining for SA-β-Gal from A. (C) The proliferation of AGS NTC and ILK-KO was measured by EdU assays. Scale bar, 100μm. (D) Number of EdU positive cells were counted in five fields. (E) Western blot analysis of several cell cycle-related proteins in the lysate with GAPDH as the loading control. (F) Oxygen consumption rate (OCR) was measured by the Seahorse analyzer in these clones with the treatment of oligomycin, FCCP and a mix of antimycin A and rotenone. (G) Basal OCR and (H) spare respiratory capacity (SRC) were determined as well. (I) Cellular ROS production was detected using a fluorescence microplate assay. (J) Extracellular acidification rate (ECAR) was measured using the Seahorse analyzer in these clones with the treatment of glucose, oligomycin and 2-deoxy-glucose (2-DG). (K) ECAR and (L) glycolysis reserve were determined from E. (M) Lactate production was measured in the culture medium of these cells. Real-time qPCR analysis of metabolism including genes regulating OXPHOS, genes encoding mitochondrial proteins (N) and genes regulating glycolysis (O) in AGS NTC and ILK-KO. (P) Flow cytometry analysis of cell cycle distribution in these selected populations. And the percentage of three cell cycle phases were measured and compared among two groups (Q)


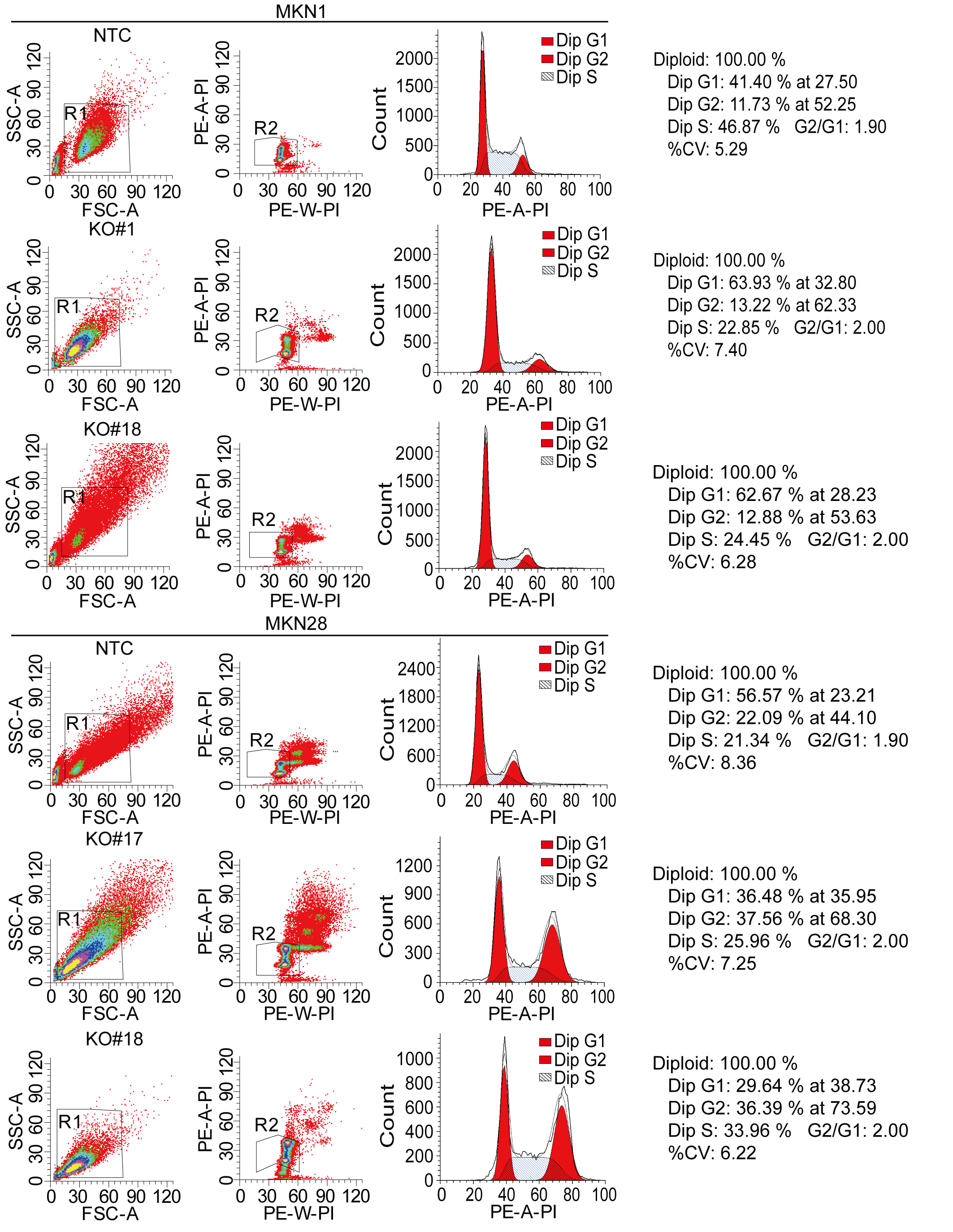


**Supplementary Figure S10. Flow cytometry analysis of cell cycle distribution in the cell lines indicated.**

Diagrams of flow cytometry analysis of cell clones of MKN1 and MKN28 with the display of forward scatter (FSC-A) and side scatter (SSC-A) to gate the live cells (R1) in the left column. In the middle column, PE-W and PE-A were used to eliminate cell doublets and cells in R2 were used for further analysis in the right column. The percentage of every cell cycle phase was shown next to the graphs.


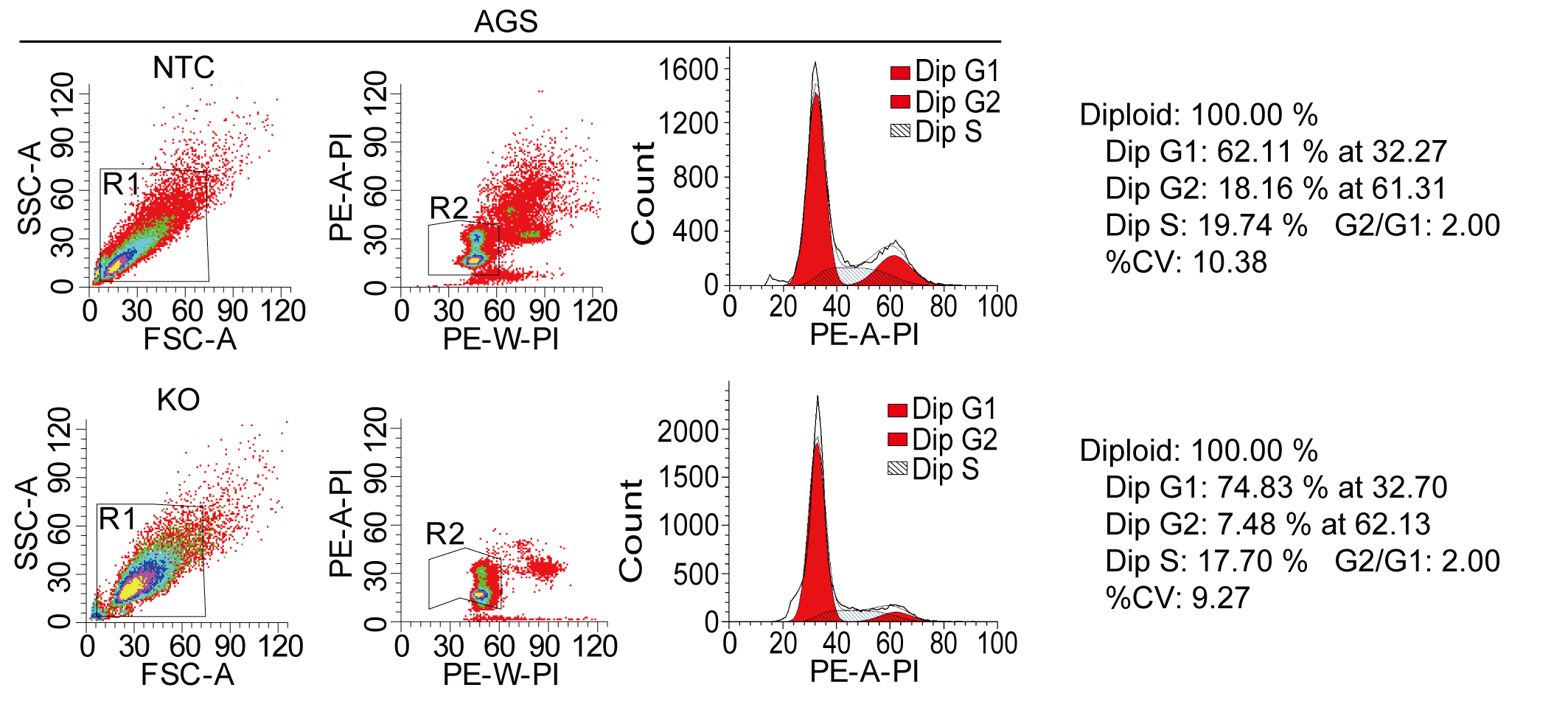


**Supplementary Figure S11.** **Flow cytometry analysis of cell cycle distribution in AGS clones.**

Diagrams of flow cytometry analysis of cell clones of AGS with the display of forward scatter (FSC-A) and side scatter (SSC-A) to gate the live cells (R1) in the left column. In the middle column, PE-W and PE-A were used to eliminate cell doublets and cells in R2 were used for further analysis in the right column. The percentage of every cell cycle phase was shown next to the graphs.

**Supplementary Table S1. Primer sequences used for SYBR qPCR gene expression assays.**

| gene | forward | reverse |
| --- | --- | --- |
| S100A8 | ATGCCGTCTACAGGGATGACCT | AGAATGAGGAACTCCTGGAAGTTA |
| S100A9 | GCACCCAGACACCCTGAACCA | TGTGTCCAGGTCCTCCATGATG |
| SERPINA1 | TCTGAAGAGCGTCCTGGGTCAA | GATGGTCAGCACAGCCTTATGC |
| VCX | GGAGGCAGGAAAGAGGAAGT | CGTCTCCCTCTACGAACTGC |
| SPANXB1 | GCCAATGAGGCCAACAAGAC | ATTCTGTTCTCTCGGGCGTG |
| 18S | CGGCTACCACATCCAAGGAA | GCTGGAATTACCGCGGCT |
| MT-ATP6 | GCACAGTGATTATAGGCTTTC | CCTGCAGTAATGTTAGCGGT |
| MT-COX1 | CAGGTTGAACAGTCTACCCT | AAGAGGGGCGTTTGGTATTG |
| MT-CYTB | ATGACCCCAATACGCAAAACT | GGGAGGACATAGCCTATGAA |
| MT-ND1 | CTACTCCTCATTGTACCCAT | GTGAAGAGTTTTATGGCGTC |
| HIF1A | ATCACCCTCTTCGTCGCTTC | ACTTATCTTTTTCTTGTCGTTCGC |
| HK2 | TCCAGAGGAGAGGGGACTTT | TCATCGCCTTCCACCATGTC |
| PFKFB3 | CCCTTCAGGAAAGCCTGTGG | GAACACTTTTGTGGGGACGC |
| PKM2 | AATGCAGTCCTGGATGGAGC | CAAGTGGTAGATGGCAGCCT |
| CREB1 | ACCAAGTTGTTGTTCAAGGTACT | ACATGTTACCATCTTCAAACTGACG |
| PRARGC1A | TCTGAGTCTGTATGGAGTGACAT | CCAAGTCGTTCACATCTAGTTCA |
| TFAM | CGCTCCCCCTTCAGTTTTGT | CCAACGCTGGGCAATTCTTC |
