# Supplementary figures and images for: The kinase activity of integrin-linked kinase regulates cellular senescence in gastric cancer
Source: Cell Death Dis. 2022 Jul 1;13(7):577. doi: 10.1038/s41419-022-05020-3 (PMC9249761; doi:10.1038/s41419-022-05020-3)

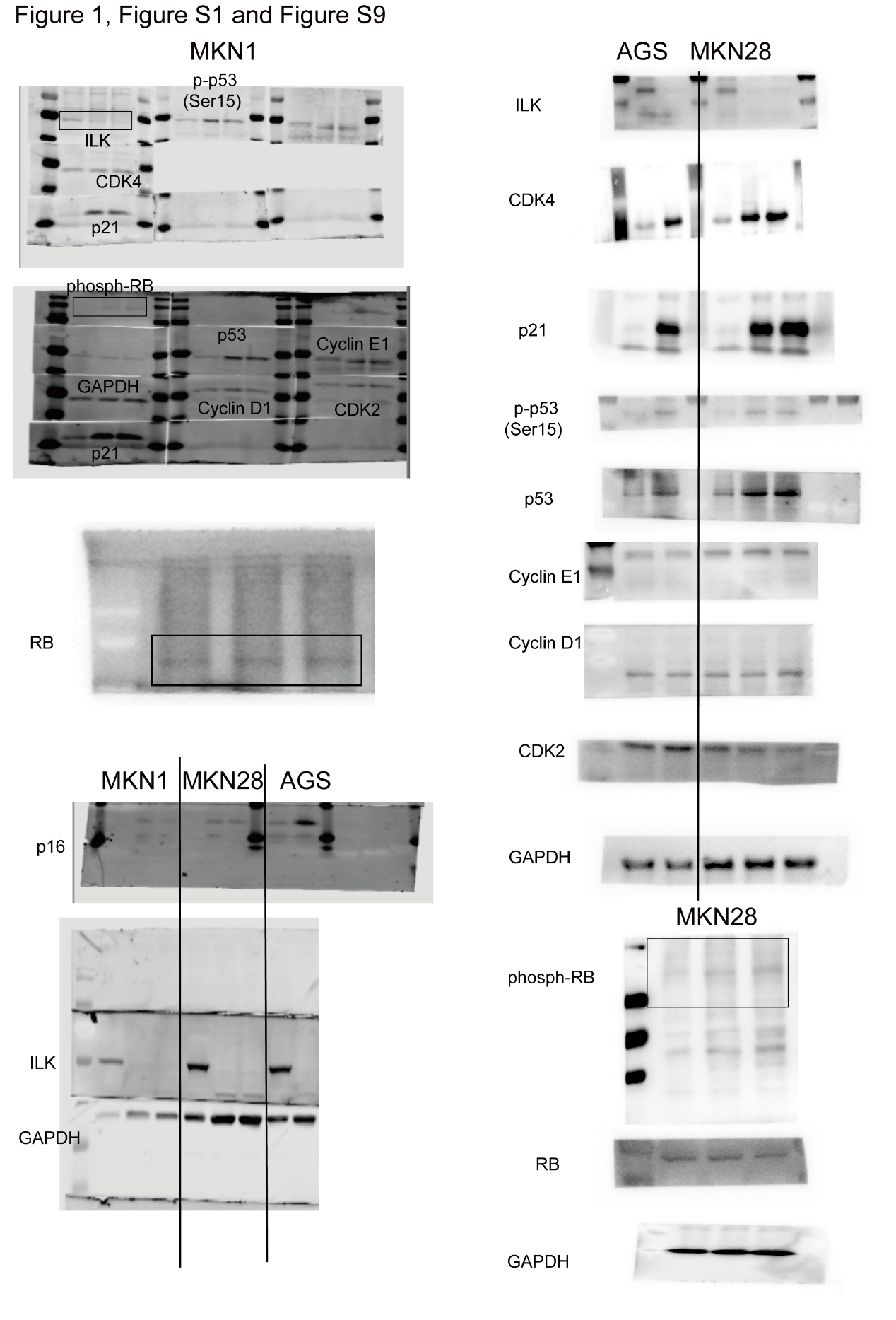


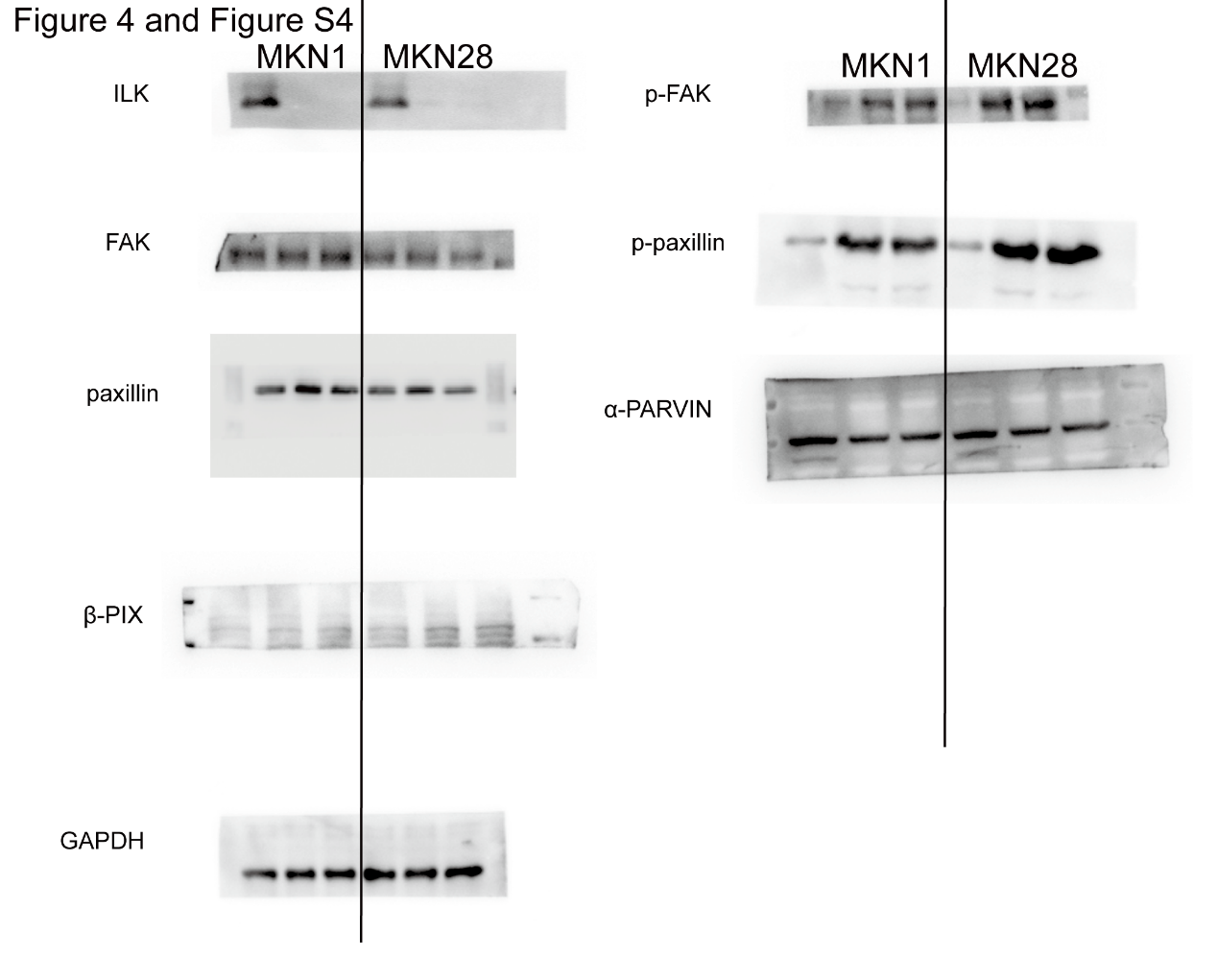

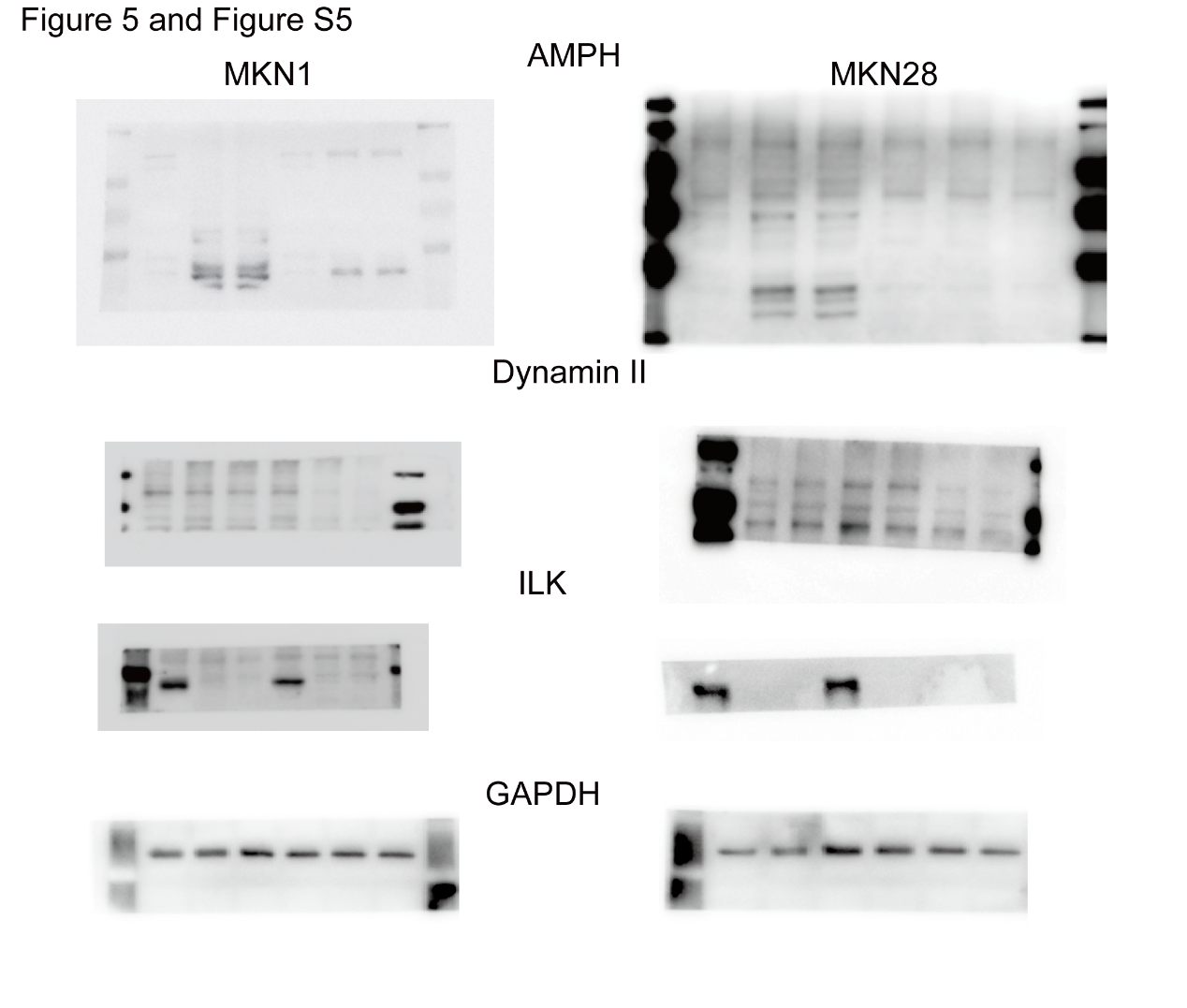

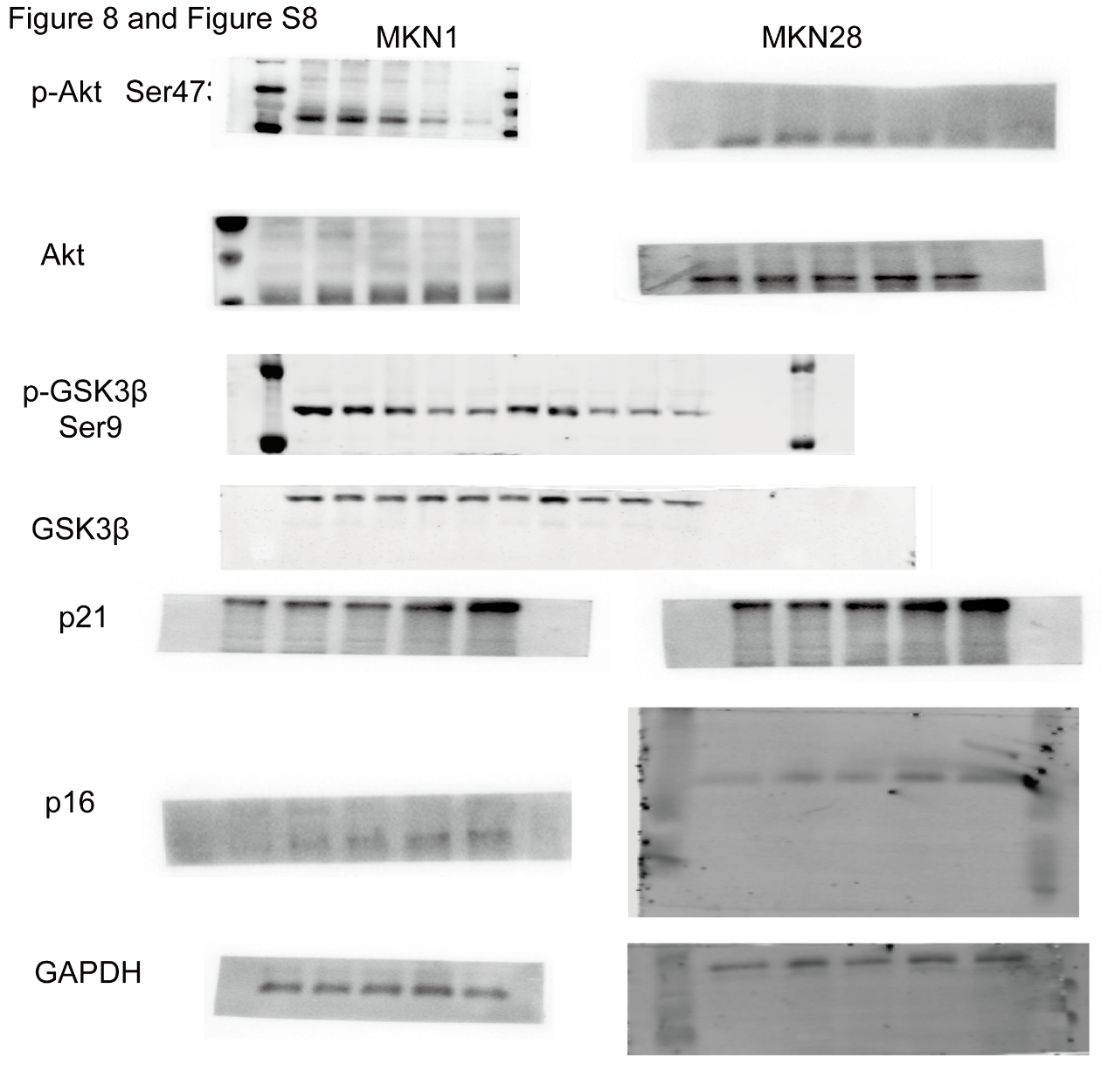

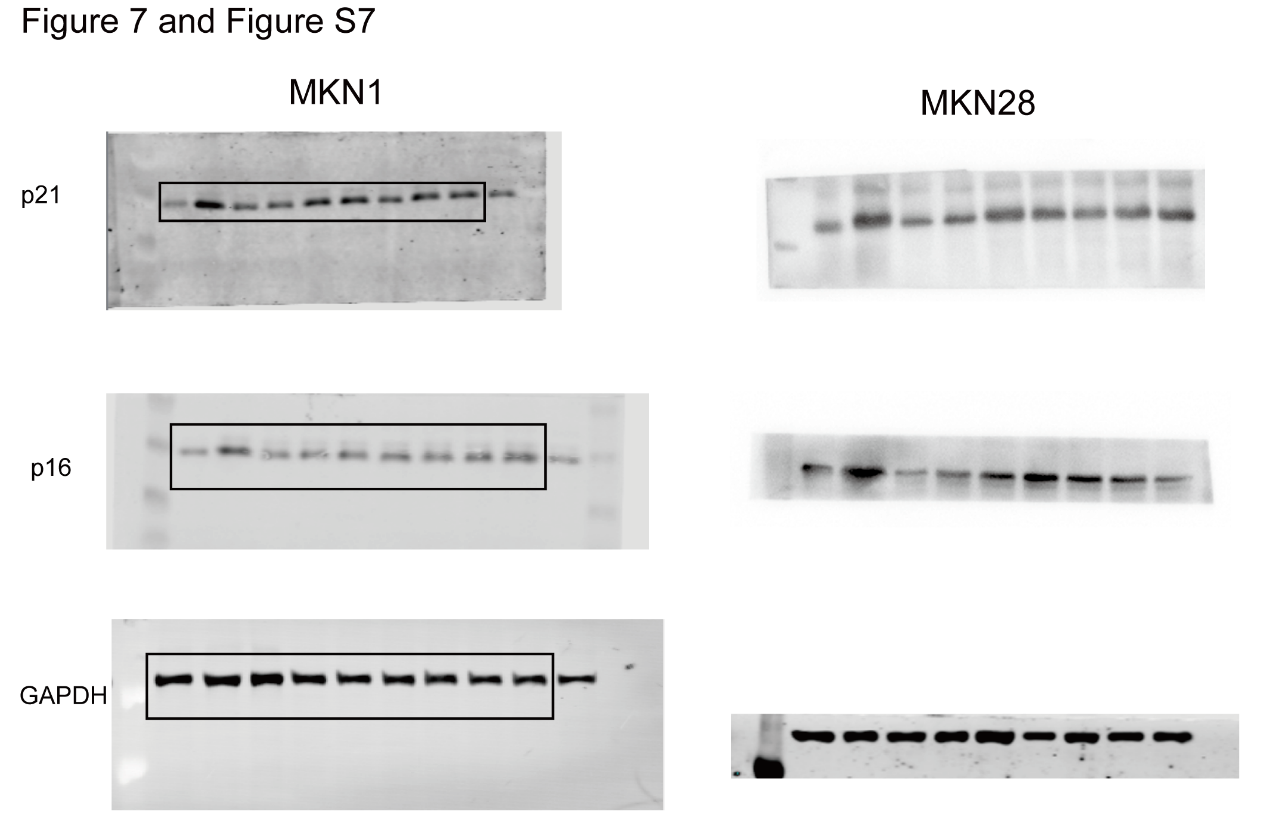

Supplement: Supplementary file 2 — Western blot Original Data File [file 41419_2022_5020_MOESM2_ESM.docx]
